# Sensor-Informed Probabilistic Bridge Reliability Analysis: A Review

**DOI:** 10.3390/s26196199

**Published:** 2026-09-30

**Authors:** Isaac Ojonogecha Adama, Ying Zhuo, Woubishet Zewdu Taffese, Genda Chen

**Affiliations:** Department of Civil, Architectural and Environmental Engineering, Missouri University of Science and Technology, Rolla, MO 65401, USA; iakqr@mst.edu (I.O.A.); yingzhuo@mst.edu (Y.Z.)

**Keywords:** bridge structural health monitoring, sensor data preprocessing, probabilistic reliability analysis, Bayesian model updating, remaining service life

## Abstract

This review examines the integration of sensor data into probabilistic bridge reliability analysis, covering the workflow from sensor selection and data acquisition through preprocessing, computational Bayesian inference and reliability index *β* computation to remaining service life estimation and maintenance optimization. Sensor selection is treated as a reliability engineering decision, with sensing technologies matched to the target reliability variable and governing deterioration mechanism. Sensor imperfections inherent to long-term structural health monitoring, including drift, bias, noise, missing data, synchronization errors, and calibration uncertainty, are evaluated through their propagation along the reliability analysis chain. Statistical filtering, thermal compensation, anomaly detection, and missing data imputation are examined as essential preprocessing stages for reliable downstream inference. Computational Bayesian methods are organized into core posterior inference techniques, including Markov Chain Monte Carlo, Sequential Monte Carlo, variational inference, approximate Bayesian computation, and neural simulation-based inference, and supporting techniques, including surrogate-based approaches, deterministic filtering, asymptotic approximations, and hybrid Bayesian reliability frameworks. The synthesis indicates that sensor imperfections can propagate into reliability indices and RSL estimates, that preprocessing is reliability-critical, and that no single inference method is universally suitable for sensor-informed bridge reliability assessment. This propagation is demonstrated by the illustrative scenario, in which RSL effects followed the ordering drift > missing data > bias; however, this ordering is conditional on the assumed parameter values and monitoring conditions and does not represent a general ranking of sensor imperfections. A key gap concerns the sensor-to-reliability pathway, as monitoring observations do not directly determine *β* but require mapping to limit state variables with consideration of identifiability and spatial representativeness. Integrating these elements with RSL estimation, value-of-information analysis, and lifecycle maintenance optimization supports evidence-based bridge management while highlighting challenges in sensor quality, model adequacy, uncertainty propagation, and computational implementation.

## 1. Introduction

Bridges are essential to transportation networks and economic stability, yet many are aging and deteriorating under increasing loads and environmental exposure [1]. In the United States, the 2025 ASCE Infrastructure Report Card reports over 621,218 bridges handling approximately 4.9 billion trips daily, of which 6.8% are in poor condition and the average bridge age is approaching its 50-year design life [1]. Although the Infrastructure Investment and Jobs Act provided $40 billion for bridge programs, a system rehabilitation need of $191 billion remains a structural funding gap that cannot be closed by capital investment alone without more rational, data-driven asset management [1]. Deteriorating bridge stocks present equivalent challenges across Europe, where the assessment of existing structures is governed by ISO 2394 [2], ISO 13822 [3], and fib Bulletin 80 [4] at the normative level. Italy has developed the most comprehensive national regulatory response through Ministerial Decrees DM 578/2020 [5] and DM 204/2022 [6], which introduced a multi-level framework for bridge risk classification, safety assessment, and monitoring that formally embeds structural monitoring, including satellite interferometric data, into the national management process. To ensure safety and functionality under these constraints, bridge assessment must move beyond episodic visual inspections toward reliability-based, data-informed preservation strategies [7].

Traditional bridge reliability analysis employs three categories of methodologies. First, approximate analytical methods, such as the first-order reliability method (FORM) [8,9] and second-order reliability method (SORM) [10,11], provide efficient failure probability estimates for relatively simple limit states. Second, surrogate modeling techniques such as response surface methods [12], including neural networks [13], Kriging model [14] and support vector machines [14], approximate complex structural responses at reduced computational cost. Third, numerical simulation methods, such as Monte Carlo simulation [15], subset simulation [16], and importance sampling [17], offer flexible but computationally intensive reliability estimates. These methods provide a probabilistic framework to quantify failure risks by accounting for uncertainties in loads, materials, and geometry [18]. However, they rely on assumed or design-stage parameters that may not reflect real-time structural conditions or evolving damage states [19,20], a limitation that becomes increasingly consequential as bridge assets age beyond their design assumptions.

The emergence of structural health monitoring (SHM) systems has transformed bridge management by enabling continuous, high-resolution data collection through sensors including strain gauges, accelerometers, fiber optic sensors, and corrosion detectors [21]. Building directly on the traditional reliability methods, SHM data enables explicit integration of field evidence into probabilistic reliability frameworks. Reliability indices, failure probabilities, and remaining service life predictions are informed by measured structural behavior rather than purely assumed design-stage models [22,23]. The principal mechanism enabling this integration is Bayesian updating, which combines prior parameter distributions with likelihood functions constructed from sensor observations to yield posterior distributions that concentrate probability on parameter values consistent with both prior knowledge and field evidence. Sequential application of this mechanism, updating the posterior as each new batch of monitoring data arrives, supports a continuous reliability assessment paradigm that can reduce epistemic uncertainty and improve predictive accuracy [22,24]. This data-informed reliability paradigm can enhance safety and enable more rational maintenance planning and lifecycle cost optimization by grounding decisions in the actual, monitored condition of the structure rather than assumed deterioration trajectories [24,25].

However, the utility of sensor-informed reliability analysis depends critically on the quality and treatment of the underlying data. Bridge SHM systems generate continuous streams of sensor data that are routinely affected by sensor drift, bias, noise, missing records, temperature effects and operational variability, and communication failures [21,26]. These imperfections do not merely reduce data quality; they propagate through the entire reliability analysis workflow. A fundamental distinction therefore applies: raw SHM data constitutes sensor observations subject to these imperfections, whereas reliability-relevant information requires preprocessing and uncertainty characterization before it can legitimately enter a likelihood function. A biased sensor record produces a biased load-effect distribution, which in turn produces a biased likelihood function, a biased posterior distribution of structural parameters, and ultimately erroneous reliability index and remaining service life (RSL) projection. Despite this, existing reviews of bridge SHM [27] and structural data science [28] treat preprocessing primarily as a data management concern rather than a reliability analysis concern. The systematic chain from specific sensor imperfections through preprocessing decisions to reliability index errors has not been comprehensively examined. This gap motivates the present review, which explicitly links sensor data quality and preprocessing to the reliability analysis workflow across the complete pipeline from sensor acquisition through Bayesian model updating to remaining service life estimation. Table 1 provides a structured comparison of the present review with key prior reviews, supporting the authors’ assessment that, to the best of their knowledge, no existing review integrates all these stages within a single sensor-to-reliability framework.

This narrative review followed a structured and iterative literature-identification process. Relevant literature was initially identified through searches of the Scopus and Web of Science databases, covering publications from 2000 to 2026. The search strategy combined terms and their relevant variants related to bridge structural health monitoring, Bayesian model updating, sensor data preprocessing, probabilistic reliability, remaining service life (RSL), satellite-based/InSAR monitoring, operational modal analysis, optimal sensor placement, digital twins, non-stationary signal processing, stochastic degradation, and POMDP-based inspection. A total of 1119 records were initially identified. Following duplicate removal, 550 records remained for title and abstract screening. Publications were considered relevant when they addressed bridge assessment or provided methodological contributions related to sensing and monitoring, sensor data preprocessing, probabilistic model updating, reliability and RSL assessment, or lifecycle inspection and maintenance decision-making. Records lacking relevance to these areas were excluded. Database searching was supplemented by backward citation tracking to identify relevant foundational and methodological sources. Seminal publications predating 2000 were additionally retained where necessary to establish the theoretical foundations of the methodological approaches discussed in the review. Following screening and subsequent refinement of the literature base, 200 publications were included in the final narrative synthesis. The selected literature was synthesized thematically according to the major methodological components of the review.

As summarized in Table 1, existing review papers typically focus on individual components of the sensor-informed bridge assessment process, such as SHM technologies, sensor data preprocessing, or Bayesian reliability methods. To the authors’ knowledge, none provides an integrated review of the complete workflow linking sensor data quality to probabilistic reliability assessment and decision-making. This paper aims to address that gap by providing an integrated conceptual synthesis of the complete sensor-to-reliability pipeline, from sensor data acquisition and quality assessment through preprocessing, Bayesian model updating, reliability index estimation, RSL estimation and maintenance optimization. Unlike prior reviews that treat preprocessing primarily as a data management concern, this review treats sensor imperfections as reliability-critical inputs and explicitly traces their propagation through every stage of the pipeline into reliability indices and RSL estimates. Emphasis is placed on computational Bayesian methods for sensor-informed bridge reliability, organized into core posterior inference techniques and the supporting computational methods that enable their practical implementation. In addition, sensor data preprocessing is treated as an essential prerequisite, with comprehensive coverage of common sensor imperfections, correction methods, and uncertainty propagation, recognizing that the quality of the input data directly influences the accuracy and validity of the reliability assessments.

The remainder of this paper is organized as follows. Section 2 presents sensor data acquisition and preprocessing for bridge reliability analysis. It begins with an overview of sensor technologies, followed by a detailed examination of common data imperfections and their impact on reliability assessments, and concludes with preprocessing and correction methods that transform raw measurements into probabilistically sound inputs. Section 3 reviews Bayesian computational methods for sensor-informed bridge reliability analysis, covering both core posterior inference methods and the supporting inference techniques that make them operationally feasible for large-scale bridge applications. Section 4 explores how these reliability assessments extend to RSL estimation, reliability-centric maintenance planning, and lifecycle decision-making. Finally, Section 5 synthesizes key findings, discusses their implications for bridge management, and identifies future research directions in sensor-informed probabilistic bridge reliability analysis.

## 2. Sensor Data Acquisition and Preprocessing for Reliability Analysis

**Figure 1:**
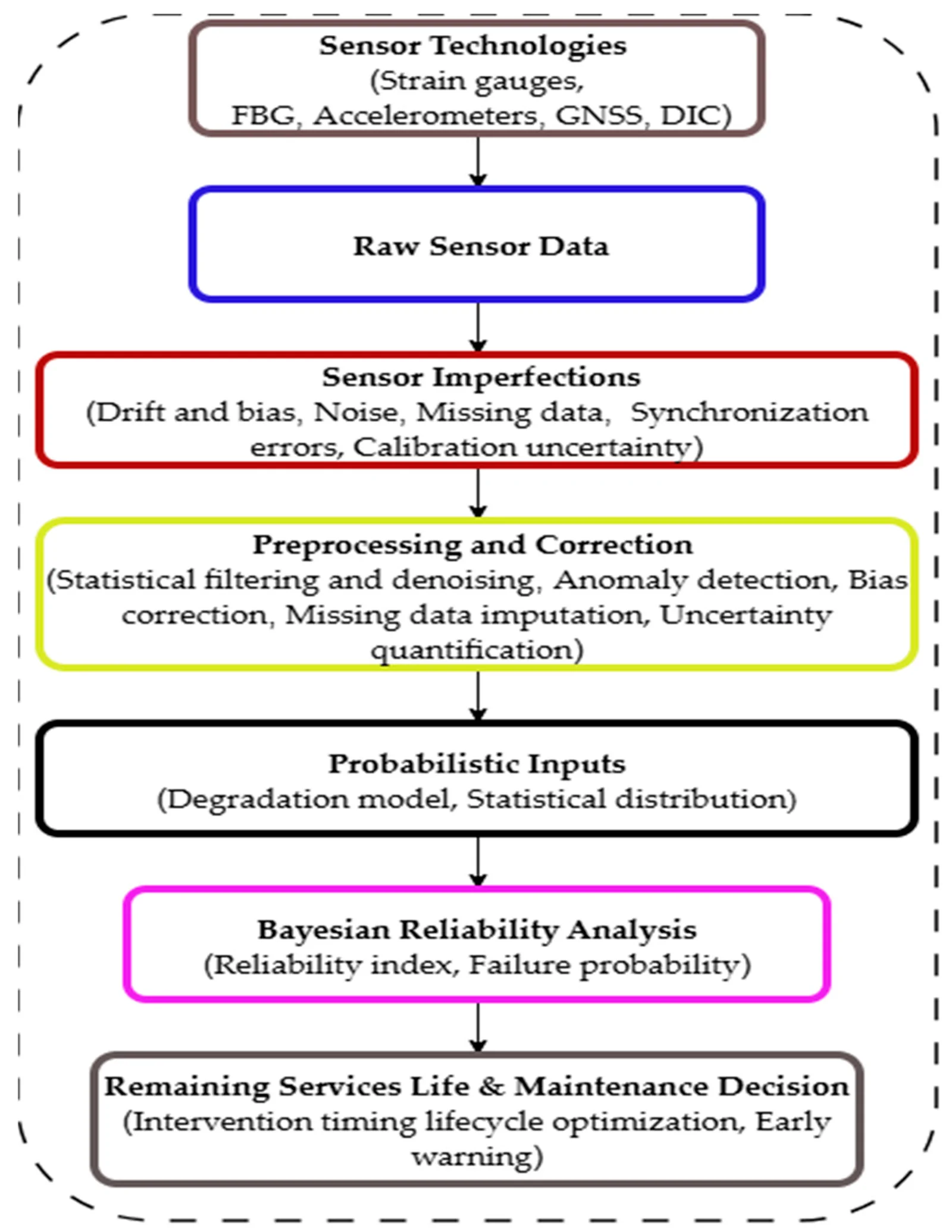
Overview of the sensor-informed bridge reliability analysis pipeline. Created by the authors.

Reliability analysis of bridge structures fundamentally depends on accurate, representative input data. The transition from traditional design-stage assumptions to sensor-informed probabilistic models requires a robust framework for data acquisition, quality assessment, and preprocessing. In bridge SHM, diverse sensing technologies, including strain gauges, accelerometers, displacement transducers, fiber Bragg grating sensors, and Global Navigation Satellite System (GNSS) receivers are widely employed to capture structural responses under operational and extreme loading conditions [21]. Appropriate sensor selection, placement, and calibration are prerequisites for generating data suitable for reliability and performance evaluation throughout the bridge lifecycle [21,33]. The complete workflow is summarized in Figure 1, which shows the sequential stages from raw sensor data acquisition through imperfection characterization, preprocessing and correction, probabilistic Bayesian updating, and reliability index (*β*) computation to RSL estimation and maintenance optimization.

### 2.1. Types of Sensors and Data

Modern bridge SHM systems employ a wide range of sensing technologies to capture both structural responses and environmental actions over the service life of the structure. The choice of sensor type is governed by the monitored parameter, required accuracy and sampling rate, site-specific environmental conditions, power and communication constraints, and long-term durability and maintainability [21,33]. Table 2 summarizes the main classes of sensors used in bridge applications together with the quantities they measure, the latent reliability variables they inform, the transfer models required, temporal and spatial resolution, principal error sources, and validation needs for bridge reliability assessment. Together these sensor technologies provide the full range of structural variables required by probabilistic reliability analysis. No single sensor type is universally optimal; effective bridge reliability assessment typically requires a combination of sensor types tailored to the target reliability variable and the governing deterioration mechanism.

### 2.2. Sensor Selection for Bridge Reliability Applications

A fundamental distinction for sensor selection is whether the dominant uncertainty in the reliability assessment resides on the load-effect side, (*S*(*t*)), or the resistance side, (*R*(*t*)). S-dominated reliability problems, such as fatigue assessment and overload detection, require high-rate, drift-compensated sensors because the load-effect probability density function (PDF) must accurately capture extreme events. Missing or underestimating these events can bias the tail of the load-effect distribution and therefore *β* [64,65]. In contrast, *R*-dominated problems, including prestress loss, corrosion-induced section loss, and stiffness degradation rely primarily on long-term measurement stability to track gradual deterioration, making long-term sensor stability more critical than high sampling rates [51,52].

Selecting an inappropriate sensor can introduce unnecessary uncertainty into the reliability analysis. For example, using accelerometers for fatigue reliability assessment produces an indirect *β* estimate with additional uncertainty from model inversion [66,67], whereas direct strain measurement provides the empirical load-effect PDF required for FORM-based reliability computation [65,68]. Conversely, employing strain gauges for prestress loss assessment in concrete bridges would require impractical long-term baseline stability, whereas deflection transducers capture the integrated long-term deformation that reflects creep, shrinkage, prestress loss, and support effects collectively; separating individual contributions requires a calibrated structural model [51,52]. Satellite radar interferometry (InSAR) constitutes a distinct sensing modality that requires no on-structure installation and provides retrospective wide-area displacement histories, serving as a preliminary network-scale information layer that can inform prior distributions or prioritization for subsequent bridge-specific Bayesian updating [56,57,58]. Regardless of sensor type, sensors observe noisy proxies of structural response rather than resistance or load-effect distributions directly. A forward model linking the measured quantity to the limit-state variable together with identifiability assumptions and spatial representativeness is required before observations can enter a reliability calculation. Table 2 identifies the latent reliability variable and transfer model required for each sensing modality.

Beyond sensor type selection, the placement of sensors within the structure determines the information content of the monitoring system. Papadimitriou [69] established information entropy as the standard optimality criterion for structural identification. Yuen et al. [70] extended the entropy-based framework to ambient excitation without measured input, which is the standard condition in bridge SHM. Kammer [71] introduced the effective independence method, which maximizes the determinant of the FIM iteratively to preserve the linear independence of target mode shapes across sensor locations. Papadimitriou and Lombaert [72] showed that spatially correlated prediction errors inflate the apparent information content of co-located sensors and must be accounted for to avoid suboptimal placement. Together these methods provide the theoretical toolkit for designing bridge monitoring systems that are optimally informative for reliability assessment. Others bridge-specific applications include sensitivity-based strain gauge placement for damage identification of steel truss bridges [73] and partial-model-based damage identification of long-span steel truss bridges using stiffness separation [74].

These trade-offs including preprocessing requirements, measurement directness, and long-term stability must be evaluated against the specific reliability problem, available resources, and required accuracy of the reliability index *β*, as summarized in Table 2. The computational Bayesian methods governing all these sensor pathways are the primary subject of this review and are examined in detail in Section 3.

### 2.3. Sensor Imperfections and Their Impact on Reliability

The sensor technologies reviewed in Section 2.1 are capable of capturing the load effects, resistance parameters, and deterioration indicators that reliability analysis requires but only if their data are of sufficient quality. In practice, however, real-world bridge deployments are subject to sensor imperfections that degrade measurement quality before the data enters the reliability analysis workflow. If unaddressed, these imperfections propagate through subsequent analyses, leading to biased or unconservative estimates. The effects of sensor imperfections extend throughout the entire reliability assessment pipeline.

Common sources of sensor data degradation in bridge SHM include missing data, bias and drift, measurement noise, synchronization errors, and calibration uncertainty [26,75]. Each of these imperfections affects reliability analysis differently and therefore require appropriate preprocessing and correction strategies. Understanding the characteristics of these imperfections, their influence on probabilistic reliability assessment, and the corresponding preprocessing methods is the focus of this section.

#### 2.3.1. Bias and Drift

Bias denotes a constant offset, $\delta_{b}$, between the sensor output and the true value, typically arising from miscalibration, while drift describes a progressive deviation, $\delta_{d}\left(t\right)$, over time due to environmental exposure, sensor aging or material degradation [76,77]. In one SHM study of a pedestrian bridge, drift constituted nearly 75% of identified sensor faults in accelerometers [76], suggesting that drift may be among the most prevalent fault types in long-term deployments. Consequently, both bias and drift are recognized as major sensor fault types in SHM systems requiring explicit fault detection and correction strategies rather than conventional passive filtering techniques [76]. Wang et al. [78] addressed online sensor fault detection using meta-learning with limited training data, demonstrating applicability to SHM systems.

Drift is particularly critical in long-term SHM deployments because it requires explicit modelling or drift-correction strategies; passive approaches that work for random noise cannot remove a systematic time-varying offset [79]. In bridge reliability applications, systematic bias and drift consistently distort estimates of load effects, environmental actions, and deterioration rates. If these measurements are assumed to be unbiased, the resulting errors propagate through Bayesian updating, leading to biased value-of-information estimates [79], reliability indices and RSL projections. Unlike random measurement noise, the impact of drift accumulates throughout multi-year monitoring campaigns making it one of the most critical sources of uncertainty in sensor-informed reliability assessment over the very timescale on which deterioration trends and RSL are assessed.

A key practical challenge in long-term monitoring is distinguishing true sensor drift from genuine structural changes that produce similar slowly evolving trends. For example, creep and shrinkage in concrete bridges cause progressive strain and deformation trends that closely resemble drift, while corrosion-induced section loss in steel bridges progressively alters structural stiffness and load–response relationships [7]. To differentiate these effects, physics-based models of expected long-term structural behavior can be combined with signal decomposition methods that separate creep, shrinkage, temperature, and live load components from the measured signals [80]. The remaining residual component can then be attributed to sensor drift, enabling appropriate correction before reliability analysis.

#### 2.3.2. Noise

Noise appears as high-frequency random fluctuations with variance ($\delta_{\varepsilon }^{2}$) superimposed on the true signal, originating from electrical interference, mechanical vibration, and non-ideal signal processing [76]. Unlike systematic bias or drift, noise is typically modelled as random measurement deviations that cannot be eliminated entirely but can be substantially reduced through preprocessing such as filtering and wavelet-based denoising [76]. In bridge SHM, environmental and operational conditions frequently contaminate data with noise, obscuring genuine structural responses and hindering reliable anomaly detection and automated structural warning [81].

From a reliability perspective, the impact of noise on reliability assessment is asymmetric: if the additional variance from noise is not propagated into the reliability model, reliability index (*β*) appears more precise than the data warrant the model is overconfident; if noise is propagated without reduction, *β* appears worse than warranted, producing overly conservative maintenance decisions. Unlike drift and bias, however, noise primarily inflates variance without systematically shifting the mean of the measure response, making it more influential on the precision than the direction of reliability estimates. For example, wavelet-based denoising methods applied to strain measurements from steel bridge decks have been shown to improve stress-range estimation and, consequently, fatigue reliability assessments [82].

#### 2.3.3. Missing or Incomplete Data

Missing data is among the most frequently reported sensor data problems in bridge SHM, arising from sensor faults, battery depletion, unstable wireless links, congestion, and environmental obstructions [83]. In probabilistic reliability analysis, missing data reduces the number of observed samples ($N_{obs}$) available for likelihood evaluation and can bias Bayesian updating when the missingness is systematic. Missing data during high-load conditions is among the most critical imperfections for bridge reliability assessment, because these observations govern the tail of the load-effect distribution and therefore strongly influence the reliability index *β*. Bridge-specific reconstruction methods, including Bayesian dynamic regression [84], Generative Adversarial Network (GAN)-based imputation [85], and Wasserstein Generative Adversarial Imputation Network (WGAIN)-based imputation [86], have demonstrated that accurate data reconstruction improves the reliability of monitoring-derived parameters used in probabilistic bridge assessment, though these results are dataset-specific and should be treated as evidence-limited rather than general conclusions. Dalmasso et al. [87] applied Gaussian process regression (GPR)-based imputation to bridge SHM, addressing both MAR and MNAR gaps in natural frequency records of the KW51 rail bridge.

#### 2.3.4. Synchronization Error

Synchronization errors arise from temporal misalignment between sensors and primarily affect bridge SHM applications relying on multi-sensor data fusion, such as modal analysis, load attribution, and combined Weigh-in Motion (WIM)-strain monitoring. Synchronization is widely recognized as one of the primary challenges in bridge SHM, alongside data quality, bandwidth management, and structural condition interpretation [88]. Asynchronous or irregular sampling can mimic or mask true changes in structural behavior, distorting reliability indicators that rely on variance or autocorrelation analyses. Because relationships among structural responses are inferred from temporally aligned sensor measurements, synchronization errors produce incorrect load-effect correlations and may lead to spurious detection of structural changes or failure to identify true anomalies. Consequently, they can propagate into probabilistic reliability analyses, producing inaccurate estimates of structural reliability. Their impact is particularly significant in multi-sensor fusion applications, particularly those combining WIM load measurements with structural strain responses, where temporal misalignment between the applied loads and measurements with structural strain responses directly biases the estimated load-effect relationship. In contrast, synchronization errors have relatively limited influence on reliability assessments based on single-sensor stream.

#### 2.3.5. Calibration Uncertainty

Even after calibration, sensors retain residual systematic deviation ($\delta_{c}$) and random uncertainty arising from imperfections in calibration procedures and reference standards. During long-term bridge monitoring, sensors’ performance gradually deteriorates because of mechanical fatigue, temperature and humidity cycles, and electronic component ageing, causing calibration accuracy to degrade over time [77]. Yang et al. [77] document quantified examples of this progressive accuracy loss, confirming that calibration degradation is an operational reality in bridge SHM rather than a theoretical concern.

A practical challenge in long-term monitoring is the trade-off between recalibration and data continuity. Dismantling sensors for recalibration interrupts continuous monitoring and disrupts the historical reference baseline, while leaving sensors in service allows calibration errors to accumulate [77]. This issue is particularly important for deformation and strain sensors, whose measurements represent incremental accumulation relative to an initial reference state. Consequently, sensor replacement or recalibration alter the load-effect distributions that underpin probabilistic reliability models [77]. When residual calibration bias is ignored or treated solely as random noise, reliability assessments systematically misstate the probability of exceeding limit states, and sensor-informed safety assessments may assign unwarranted confidence to flawed observations [89]. While bias and drift are addressed earlier in this section, it is worth clarifying their relationship to calibration uncertainty. Bias and drift both originate from calibration imperfections; bias as a fixed installation-time offset, and drift as a time-dependent calibration error that develops progressively through environmental exposure and material aging [77]. Calibration uncertainty is distinct: it refers to the irreducible imprecision inherent in the calibration procedure and reference standards used during sensor commissioning, which persists as a residual uncertainty budget even after the calibration process is completed [77].

The propagation of drift, bias, and missing data under the MNAR mechanism into *β* and RSL can be illustrated through a simple limit-state calculation using Equations (5) and (7). Consider a bridge girder with normally distributed resistance *R* and load effect *S*, with ${µ}_{R}=5.0MN$, $\sigma_{R}=\;0.75MN(CoV=0.15)$, ${µ}_{S}=2.5MN$, and $\sigma_{S}=\;0.50MN(CoV=0.20)$, giving *β*_0_ = 2.77 and RSL_0_ = 8.0 years at $\beta_{target}=2.5$ and deterioration rate *α* = 0.034 per year. Sensor drift at $\delta_{d}$% per year reduces the apparent mean load effect to ${µ}_{S,app}=\;{µ}_{S}-\;{µ}_{S}.\;\delta_{d}T$ after *T* years of undetected drift; sensor bias of $\delta_{b}$% reduces it to ${µ}_{S,app}=\;{µ}_{S}-\;{µ}_{S}.\;\delta_{b}$. Both affect only the apparent mean, leaving $\sigma_{S}$ unchanged. Negative errors (underestimate load effect) produce unconservative assessments; positive errors (overestimate load effect) may trigger unnecessarily early maintenance. Both directions are plausible depending on sensor type and installation. MNAR missing data that removes the top 10% of load events truncates the upper tail of the load-effect distribution, modifying both the apparent mean and standard deviation through the truncated normal ${µ}_{S,app}=\;{µ}_{S}-\;\frac{\sigma_{S}\varphi \left(z_{p}\right)}{\Phi \left(z_{p}\right)}$ and $\sigma_{S}=\;\sigma_{S}\sqrt{\left[1-\frac{z_{p}\varphi \left(z_{p}\right)}{\Phi \left(z_{p}\right)}-{\left(\frac{z_{p}\varphi \left(z_{p}\right)}{\Phi \left(z_{p}\right)}\right)}^{2}\right]}$, where $z_{p}$ = *Φ*^−1^(0.90), *φ*(·) is the standard normal density, and *Φ*(·) is the standard normal cumulative distribution function. In the combined scenario: ${µ}_{S,app}={µ}_{S,miss}-{µ}_{S}.\;\delta_{d}T-{µ}_{S}.\;\delta_{b}$. The updated apparent $\beta_{app}$ and ${RSL}_{app}$ follow directly from Equations (5) and (7) applied to the apparent parameters. Table 3 reports the results for $\delta_{d}$ = 2%/yr with *T* = 5 years, $\delta_{b}$ = 5%, and *p* = 10%, individually and in combination. Undetected sensor imperfections produce systematically unconservative reliability assessments and rank in severity as drift > missing data > bias for the chosen parameter values, measured by the absolute RSL change. Each imperfection delays identification of the true structural condition, postponing maintenance beyond the structurally safe threshold. This is a conditional illustration for the chosen parameter values; results should not be interpreted as evidence of universal severity rankings.

Sensor failure rates, redundancy, availability, and maintainability are system-level properties of the monitoring installation that propagate directly into the achievable *β* and RSL. High sensor failure rates reduce the number of observations available for likelihood evaluation, directly increasing posterior uncertainty in the reliability-relevant parameters [90]. Non-redundant sensor configurations lose an entire parameter direction when one sensor fails, whereas redundant configurations sustain posterior concentration even under partial sensor loss [91]. Availability determines the completeness of the load-effect record; gaps occurring during extreme loading events may constitute an MNAR mechanism when missingness depends on the unobserved response magnitude, which biases the tail of the load-effect distribution governing *β*. Maintainability determines how quickly failed sensors can be restored; poor maintainability extends data gaps and reduces total information yield over the monitoring lifetime. A monitoring system optimized for information gain without accounting for these four properties may deliver substantially less total information than its design specification suggests [90,91]. Monitoring system design should therefore integrate sensor reliability into the information-theoretic placement criterion from the outset.

#### 2.3.6. Non-Stationary and Nonlinear Analysis

Bridge modal parameters vary with temperature, traffic, wind, and structural condition, making monitoring data inherently non-stationary. This non-stationarity masks damage-induced changes and is the primary source of false alarms in data-driven SHM [92]. Standard Fourier analysis assumes stationarity and is unsuitable for tracking time-varying modal behavior. Choosing an appropriate method for the non-stationarity type present is a prerequisite for credible Bayesian reliability updating, because biased modal parameter estimates propagate into posterior distributions of structural parameters and ultimately bias the reliability index *β*. Several time–frequency methods address this: the short-time Fourier transform computes local spectra from short windows; the continuous wavelet transform provides variable time–frequency resolution; the Stockwell transform combines wavelet localization with the absolute phase reference of the Fourier transform [93]; the Hilbert–Huang transform decomposes signals through empirical mode decomposition (EMD) into physically meaningful narrow-band components suited to nonlinear and non-stationary responses; variational mode decomposition (VMD) avoids the mode-mixing problem of EMD through constrained variational optimization; and the Wigner-Ville distribution offers high resolution but is subject to cross-term interference. Time-varying ARMA models and subspace identification methods track evolving modal parameters sequentially without requiring stationarity [94].

Environmental and operational variations shift modal parameters even in the absence of damage. Cointegration, introduced to SHM by Cross et al. [95], removes common non-stationary trends to produce stationary damage-sensitive residuals. Sohn [92] reviews data normalization approaches for separating environmental from structural changes. Rainieri and Fabbrocino [96] and Reynders [94] provide the canonical treatments of operational modal analysis (OMA), which identifies modal parameters from ambient excitation without measured input. Ditommaso et al. [93] introduced the band-variable filter based on the Stockwell transform for tracking time-varying eigenfrequencies and mode shapes of non-stationary structural systems. Limongelli and Çelebi [97] address seismic SHM for bridges under strong motion.

Three identification uncertainty sources are important for bridge reliability. First, damping estimates from ambient vibration carry large coefficients of variation, often tens of percent, due to limited frequency resolution and environmental sensitivity; this must be propagated into reliability assessments [94,96]. Second, amplitude dependence of modal parameters, where stiffness decreases and damping increases with response amplitude, reflects nonlinear behavior from hysteresis, crack opening and closing, and joint slip; nonlinearity indices quantify this and these effects constitute model discrepancy that propagates into β [92,93]. Modal parameters identified under different operational conditions are not directly comparable and cannot legitimately be pooled into a single Gaussian likelihood without normalizing for operational variability [92,95]. Third, model discrepancy and identification uncertainty dominate sensor noise as error sources in vibration-based updating [31], regardless of the time–frequency method used. These identification uncertainty sources must be addressed before modal-parameter-derived reliability estimates can be considered credible inputs to Bayesian updating.

### 2.4. Preprocessing and Correction Methods

Before sensor data can be used in reliability models, it must first be cleaned, corrected, and transformed into inputs with explicit probabilistic meaning. The preprocessing pipeline encompasses several complementary stages, including statistical filtering and denoising, anomaly detection, bias and drift correction, missing data imputation, and uncertainty quantification. Each stage must preserve, rather than suppress, the uncertainty needed for downstream Bayesian updating and reliability analysis [27,28]. Table 4 summarizes the principal preprocessing methods, their applications, advantages, and limitations in bridge SHM.

#### 2.4.1. Statistical Filtering and Denoising

Statistical filtering methods, including sliding-window averaging, median filtering, and threshold-based outlier removal using the 3*σ* criterion, are among the most widely deployed preprocessing techniques in bridge SHM. Applied during data acquisition or at the edge-computing layer, these techniques remove impulsive noise and obvious sensor anomalies before the data enters the reliability analysis pipeline, thereby facilitating real-time monitoring and reducing computational demand [29]. While these methods are effective for stationary or low-noise signals, their performance deteriorates when structural responses and measurement noise overlap in the time or frequency domain.

For non-stationary strain and acceleration signals wavelet-based denoising methods provide superior performance because they preserve both temporal and frequency information. The Maximum Overlap Discrete Wavelet Transform (MODWT) has been successfully applied to strain measurements from masonry arch and steel bowstring bridges, demonstrated improved signal amplitude preservation and structural feature retention under high-intensity white-noise conditions compared with conventional time-domain or frequency-domain methods. Its performance has been evaluated using Signal-to-Noise Ratio, Peak Signal-to-Noise Ratio, Root Mean Square Error, and correlation coefficient metrics across real bridge datasets [82]. Similarly, improved wavelet-threshold denoising methods have also been applied to concrete strain measurements from the immersed tunnel of the Hong Kong–Zhuhai–Macao Bridge (HZMB), where sparse-index and coefficient-of-variation criteria were used to select the optimal wavelet basis and threshold. The denoised signals were subsequently analyzed using an AutoRegressive Integrated Moving Average (ARIMA) model for structural anomaly prediction [21,28].

Kalman-type filters provide a complementary denoising strategy by recursively estimating latent structural states from noisy sensor measurements through the optimal combination of model predictions and sensor observations while minimizing the posterior error covariance. Adaptive Kalman filtering has become a standard tool in bridge SHM for mitigating measurement noise and compensating for environmental effects in long-term monitoring data [27,29].

Despite their effectiveness, all denoising methods involve an inherent trade-off between noise suppression and signal preservation. When measurement noise overlaps substantially with the structural response, no filtering technique can completely remove noise without the risk of attenuating genuine structural information. Consequently, the choice of denoising method must be guided by the spectral and statistical characteristics of both the monitored response and the dominant noise source to preserve the integrity of the load-effect distributions used in subsequent Bayesian updating and probabilistic reliability models.

#### 2.4.2. Anomaly Detection and Outlier Handling

Bridge SHM sensors continuously collect data from strain gauges, accelerometers, deflection transducers, and WIM sensors. These measurements are routinely affected by outliers arising from sensor malfunctions, communication errors, and environmental disturbances. If undetected before entering the reliability analysis pipeline, anomalous readings distort load-effect distributions and bias the posterior parameter estimates obtained through Bayesian updating. For example, undetected trend or drift anomalies in strain measurements can systematically alter the load-effect PDF, which in turn produces biased likelihood functions, posterior distributions, and ultimately erroneous estimates of the reliability index and RSL.

The primary objective of anomaly detection in reliability-oriented bridge SHM is therefore distinguishing genuine structural responses from data-quality artefacts. Given the volume and continuous nature of long-term monitoring data, this task increasingly relies on automated algorithms capable of processing sensor streams in real time before they are incorporated into probabilistic reliability model [21,81]. Machine-learning methods, including deep learning-based classification and ARIMA-based dynamic thresholding have demonstrated strong performance in detecting and removing anomalous measurements while preserving structural information required for reliability assessment [21,81].

A representative example is the application of deep learning to acceleration data from a cable-stayed bridge, where missing data, outliers, trend, drift, and square wave anomalies were successfully identified from continuous monitoring streams, demonstrating the feasibility of multi-pattern anomaly detection for long-term bridge monitoring [81]. Similarly, an ARIMA-based hierarchical warning system applied to strain measurements from the immersed tunnel of the HZMB detected genuine structural anomalies while suppressing false alarms caused by sensor malfunctions, thereby preventing corrupted measurements from propagating into the load-effect distributions used for reliability assessment [21].

Despite these advances, anomaly detection remains inherently imperfect. For example, a deep learning framework evaluated on real bridge monitoring data reported a residual undetected anomaly rate of approximately 14.4% [81]. In anomaly detection problems where anomalous observations are rare, overall accuracy can be high even when most anomalies are missed; detection performance is better characterized by sensitivity, specificity, precision, and the operating point on the receiver operating characteristic (ROC) curve. Such residual errors represent an additional source of uncertainty that should be explicitly acknowledged and propagated through Bayesian reliability assessments rather than anomaly-free sensor data.

The alarm problem is fundamentally a hypothesis-testing and Bayes-risk problem. Every threshold choice involves a trade-off between Type I errors (false alarms) and Type II errors (missed detections). The optimal operating point on the ROC curve minimizes the expected cost of misclassification rather than overall accuracy [99]. For sequentially arriving data, Shewhart charts detect sudden mean shifts; Cumulative Sum Control Chart (CUSUM) and Exponentially Weighted Moving Average (EWMA) charts accumulate evidence across successive observations and are more sensitive to gradual drift [99]. Where the decision is sequential, the Sequential Probability Ratio Test (SPRT) of Wald [100] provides the optimal rule for prescribed Type I and Type II error rates with the minimum expected number of observations. For multi-sensor networks, individual per-sensor alarm rates at conventional significance levels produce unacceptably high system-level false alarm rates; false discovery rate (FDR) control bounds the expected proportion of false alarms among all raised alarms [101].

Extreme value theory calibrates thresholds to prescribed exceedance probabilities. The generalized extreme value (GEV) distribution and the peaks-over-threshold (POT) method, which fits a generalized Pareto distribution to threshold exceedances, connect data-driven alarm thresholds to return-period-based limits consistent with code-level reliability targets [102]. Operational alarm rules should be formulated on a posterior credible bound, for instance the five per cent credible bound, rather than the posterior mean, since a point estimate ignores parameter uncertainty and will systematically understate the alarm frequency.

A fundamental distinction exists between data-driven novelty thresholds and physics-based limit-state thresholds. Data-driven thresholds detect statistical departures from learned baseline behavior and are agnostic to structural mechanics. Physics-based limit-state thresholds are defined by structural reliability analysis in terms of a prescribed exceedance probability for a code-specified $\beta_{target}$. A triggered novelty alarm warrants investigation but does not constitute evidence that a code-level limit state has been exceeded.

#### 2.4.3. Bias Correction, Thermal Compensation, and Calibration Updating

For bridge strain monitoring, one of the dominant preprocessing challenges is separating temperature-induced apparent strain from true load-induced structural response before strain measurements can be used to drive load-effect distributions for probabilistic reliability analysis [27,77]. Daily and seasonal temperature variations can impose changes in monitored bridge responses exceeding 10%, in some cases larger than those caused by structural damage itself. Consequently, if thermal effects are not explicitly removed, they can be misinterpreted as structural responses, leading to biased reliability assessments [27].

Regression-based thermal compensation methods, which establish statistical relationships between distributed temperature measurements and structural responses and Principal Component Analysis (PCA)-based methods, are among the most widely adopted approaches for long-term bridge monitoring [27,28]. These approaches separate environmental effects from structural behavior, producing temperature-compensated measurements whose residuals more accurately represent the structural response required for load-effect distribution fitting and Bayesian reliability updating.

Bayesian calibration offers a complementary strategy by estimating sensor bias and drift using reference measurements or physics-based models, while explicitly propagating uncertainty into subsequent reliability assessments. Such approaches are particularly valuable for long-term bridge SHM because sensor accuracy progressively degrades under in-service conditions owing to environmental exposure, mechanical fatigue, and component aging [77]. Recursive and online calibration updating schemes continuously adjust sensor gain and offset, preventing systematic calibration errors over the timescales to reliability assessment and RSL prediction [77].

Although thermal compensation is most commonly associated with strain monitoring, it is equally important for other sensing modalities. Satellite InSAR data are particularly susceptible to thermal confounding. Ponzo et al. [56] demonstrated that DInSAR-SBAS data alone failed to resolve the behavior of the central span of the Ponte della Musica bridge due to rapid thermal variation, becoming interpretable only when integrated with ambient vibration measurements and a calibrated numerical model. For example, physics-informed machine learning has been successfully applied to displacement measurements of bridge bearings, where data-driven models constrained by thermal physics effectively separate environmental effects from structural behavior while enabling sub-millimetric anomaly detection [103].

Regardless of the sensing technology, bias correction, thermal compensation, and calibration updating methods share a common objective: to ensure that the processed measurements accurately represent the true structural response, together with its associated uncertainty, before they are incorporated into Bayesian reliability analysis.

#### 2.4.4. Missing Data Imputation

Missing data mechanisms follow Rubin’s taxonomy [104]: missing completely at random (MCAR), where missingness is independent of all values; missing at random (MAR), where missingness depends only on observed values; and missing not at random (MNAR), where missingness depends on the unobserved value itself. Sensor failures during extreme loading events may constitute an MNAR mechanism when the probability of missingness depends on the unobserved load or response magnitude. Imputation methods validated under MCAR or MAR assumptions will therefore produce reconstructions biased in the upper tail of the load-effect distribution that governs *β* [104].

Missing data is one of the most common challenges in bridge SHM, directly compromising the reliability of structural condition assessment and sensor-informed maintenance decisions. Consequently, accurate reconstruction of missing sensor records is an essential preprocessing step before measurements can be incorporated into reliability models [84]. For short gaps, interpolation and smoothing techniques are generally sufficient. However, accuracy degrades significantly for continuous or systematic data loss, particularly when sensor failures occur during extreme loading events that dominate the tail of the load-effect distribution and therefore have the greatest influence on the reliability indices. For longer-duration gaps and systematic missingness, model-based imputation is required, though the choice of method must be matched to the missingness mechanism. Bayesian dynamic regression (BDR) is a well-established approach that reconstructs missing bridge monitoring data by exploiting correlations among multiple sensors through Kalman filtering and expectation-maximization algorithms. Its effectiveness has been demonstrated on long-span cable-stayed bridge monitoring data [84].

More recently, deep learning methods have demonstrated superior performance for high missing-data rates and extended gaps. Representative examples include GAN-based imputation validated on the Lieshihe box-girder bridge in China [85], WGAIN-GP applied to the Tongling long-span highway–railway bridge across the Yangtze River [86], and probabilistic Bayesian tensor learning for reconstructing continuous strain and temperature fields in concrete bridges. The latter maintained acceptable reconstruction accuracy for random missing rates up to 80% and structured missing rates of up to 40% [98]. These methods are developed and validated under ignorable missingness assumptions: GAN and WGAIN-GP assume MCAR, validated by artificially deleting data at random [85,86]; BDR assumes MAR, exploiting inter-sensor correlations from observed data [84]. None is designed or validated for the MNAR case where missingness depends on the unobserved extreme values that govern *β*. For MNAR data, three approaches are available: selection or pattern-mixture models that explicitly model the missingness mechanism [104]; multiple imputation rather than single imputation, since single imputation understates variance; or handling missingness directly inside the likelihood function without imputation, which avoids reconstruction bias entirely. Where the missingness mechanism cannot be verified, sensitivity analysis across imputation assumptions is recommended.

#### 2.4.5. Uncertainty Quantification

A critical requirement of reliability-oriented bridge SHM is that each preprocessing step explicitly quantifies and propagates residual uncertainty rather than suppressing it. Neglecting uncertainties produces overconfident reliability assessments, while excessive uncertainty propagation results in overly conservative estimates. Accordingly, preprocessing should preserve the uncertainty associated with sensor measurements so that subsequent Bayesian updating accurately reflects the confidence in the available data.

Three distinct uncertainty sources should be considered during preprocessing: (i) *process uncertainty*, arising from the inherent variability of structural responses, (ii) *measurement uncertainty*, resulting from the sensor imperfections discussed in Section 2.3, and (iii) *model uncertainty*, introduced by the preprocessing algorithms themselves, including filtering, anomaly detection, and imputation [27]. These uncertainty components must be propagated through the entire reliability analysis pipeline because they directly influence the posterior distribution of structural parameters and, therefore, the precision of the reliability index *β*. For example, treating non-stationary noise as Gaussian white noise or neglecting the uncertainty associated with recovered missing values produces an artificially narrow posterior distribution and an overconfident estimate of structural safety.

Bridge SHM demonstrates that preprocessing can be designed to preserve uncertainty explicitly. The BDR framework proposed by Zhang et al. [84] jointly estimates missing sensor values and their associated uncertainty using Kalman filtering and Expectation-Maximization algorithms, while the probabilistic tensor imputation framework of Ren et al. [98] reconstructs continuous strain and temperature fields together with their uncertainty. These examples illustrate that uncertainty can be carried forward rather than discarded during preprocessing, thereby improving the reliability and credibility of downstream Bayesian inference.

Each preprocessing stage contributes a characteristic residual uncertainty that must be incorporated into the overall uncertainty budget of the processed sensor record. Statistical filtering leaves residual noise ($\sigma_{\varepsilon }^{2}$), anomaly detection retains a finite probability of undetected anomalies [81], thermal compensation contributes residual uncertainty ($\sigma_{T}^{2}$) under non-stationary temperature fields [27], missing-data imputation introduces reconstruction uncertainty ($\sigma_{imp}^{2}$) that depends on the duration and pattern of data loss [84,98], and calibration updating carries residual calibration uncertainty ($\delta_{c}$) that grows with sensor aging and long-term deployment conditions [77]. Together, these residual uncertainties define the confidence that should be associated with the processed sensor data before Bayesian updating.

**Figure 2:**
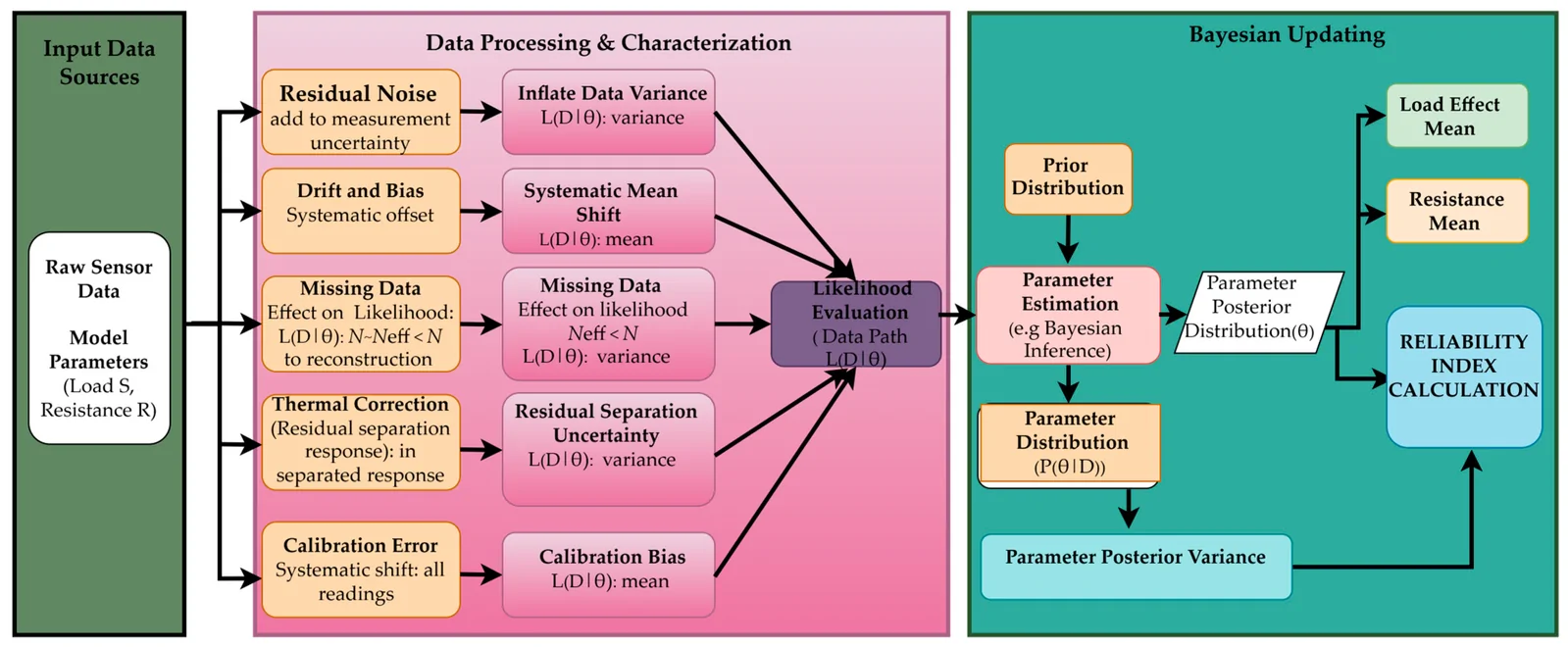
Propagation of sensor preprocessing uncertainty through the bridge reliability assessment chain. The moment-based FOSM branch is an illustrative simplification; the general posterior-predictive reliability index is defined in Section 3.2. Created by the authors.

Figure 2 summarizes sensor preprocessing uncertainty propagation chain: each imperfection type; residual noise, drift and bias, missing data, thermal correction, and calibration error enters the likelihood evaluation *L*(*D*|*θ*) and propagates through the posterior *P*(*θ*|*D*). As a FOSM illustration, the posterior moments load effect ($\mu_{S}$ and $\sigma_{S}^{2}$), resistance mean ($\mu_{R}$ and $\sigma_{R}^{2}$), feed into the reliability index β = ($\mu_{R}-\;\mu_{S}$)/$\surd (\sigma_{S}^{2}+\;\sigma_{R}^{2})$, illustrated here as a plug-in Cornell estimate; the posterior predictive $\beta_{pred.}$ is defined in Section 4.1. Here, $\sigma_{S}^{2}\;$ and $\sigma_{R}^{2}$ represent aleatory variability, not epistemic posterior variance; substituting the latter would underestimate failure probability [105]. The general posterior-predictive quantity $\beta_{pred.}$ is defined in Section 3.2.

Section 2.4 follows a clean-then-infer architecture in which sensor imperfections are corrected prior to Bayesian inference. The observation model for a raw sensor record is $D\left(t\right)={x\left(t\right)+\delta }_{d}\left(t\right)+\delta_{b}+\varepsilon \left(t\right)$, where $x\left(t\right)$ is the true structural response, $\delta_{d}\left(t\right)$ is the drift, $\delta_{b}$ is the bias, and ${\varepsilon \left(t\right)\sim N(0,\sigma }_{\varepsilon }^{2}$), is the measurement noise. In the clean-then-infer route, a preprocessing operator $f\left(.\right)$ produces $D^{\prime }\left(t\right)=f\left(D\left(t\right)\right)\approx x\left(t\right)+\varepsilon^{\prime }\left(t\right),$ which is treated as observed data in *L*($D^{\prime }$|*θ*). The two routes are equivalent only when $f\left(.\right)$ removes all systematic error leaving independent Gaussian residuals, an assumption rarely verified in practice. Otherwise, $f\left(.\right)$ may alter marginal distributions, distort temporal and cross-sensor dependence, and truncate the extreme tails governing failure probability; treating $D^{\prime }$ as error-free does not yield a coherent likelihood unless residual uncertainty is explicitly characterised. The propagation of $\delta_{d}\left(t\right)$ and $\delta_{b}$ through the observation model into $\beta_{app}$ and RSL is illustrated in Table 3. Model discrepancy, which is the mismatch between the structural model and physical reality, introduces an additional source of uncertainty beyond sensor errors; this is addressed through the Kennedy-O’Hagan discrepancy framework [106] discussed in Section 2.5. A statistically coherent alternative, the joint inference route, treats $\delta_{d}\left(t\right)$ and $\delta_{b}$ as latent parameters alongside *θ* and evaluates the full posterior ${P(\delta }_{d},\delta_{b}|D\propto L\left(D|\theta ,\delta_{d},\delta_{b}\right)P(\theta ){P(\delta }_{d}){P(\delta }_{d})$ over the raw record *D* directly [31].

### 2.5. Multi-Sensor Data Fusion

The sensor types as shown in Table 2 differ in the structural parameters they observe, the temporal resolution at which they sample, and the limit states they inform. No single sensor type constrains the full parameter space required for probabilistic reliability assessment. Multi-sensor data fusion addresses this complementarity by combining heterogeneous data streams into a single probabilistic inference; when sensor errors are uncorrelated and models are well specified, fusion reduces parameter uncertainty more effectively than any individual sensor [69,71,107], but correlated errors, model discrepancy, or sensor unreliability can negate this benefit.

The quantitative basis for this uncertainty reduction is the Fisher information matrix (FIM), which measures how strongly sensor data constrain each direction of the parameter space. `Under conditional independence and regular identifiable models, Fisher information is additive across sensors [69,71]; shared temperature effects, common calibration errors, model discrepancy, and spatially correlated prediction errors violate this condition and overestimate the information gain. This reduction is largest when sensors constrain different directions of the parameter space. In expectation and under the assumed model, expected information gain is non-negative and expected entropy decreases as sensors are fused; posterior entropy need not decrease monotonically for every realized dataset [107]. Likewise, maximizing expected entropy reduction and maximizing det (FIM) are equivalent only under local Gaussian/asymptotic approximations and suitable independence assumptions. Optimal sensor placement formalizes this principle directly by selecting sensor configurations that maximize the expected reduction in information entropy, or equivalently maximize the determinant of the total FIM, over all candidate sensor locations [69,71,107]. This framework connects multi-sensor fusion to Bayesian optimal experimental design; expected information gain is a pre-data design-stage quantity and need not equal the realized posterior uncertainty reduction for a specific dataset [69,72].

Several fusion architectures have been applied to bridge SHM. Hierarchical Bayesian models estimate shared structural parameters while preserving sensor-specific uncertainty [108]. Multi-rate and multi-resolution fusion of slow satellite displacement records with fast accelerometric data and thermal covariates require careful temporal alignment across sampling rates [56,57]. These sources enter joint likelihood only when a common reference frame and shared structural model are established; misalignment or model discrepancy degrades rather than improves joint inference. In Kalman and particle filter implementations, multi-rate fusion is handled by separating the measurement update from the time update [109,110]. Fusion of continuous SHM data with discrete inspection outcomes is handled by treating inspection results as likelihood observations that update the posterior of deterioration parameters [111].

Conflicting evidence across sensors requires explicit treatment rather than passive averaging. Redundant sensor networks enable fault isolation through cross-validation. When one sensor produces an observation inconsistent with the posterior predictive distribution of the remaining sensors, it is flagged as faulty rather than incorporated into the reliability update [75,76]. Heterogeneous sensing reduces identifiability constraints because sensors of different types constrain different directions of the parameter space [69,108].

Sensor reliability, including failure rates, availability, and maintainability, propagates into the achievable reduction in *β* uncertainty and RSL projections. A monitoring system with high sensor failure rates provides less informative data than its design suggests, reducing the expected value of information (VoI). Monitoring system design should account explicitly for sensor reliability alongside the FIM-based information gain criterion [69,72].

## 3. Reliability Analysis Methods Enhanced by Sensor Data

Conventional structural reliability frameworks founded on FORM, SORM, Monte Carlo simulation, and related methods generally rely on assumed probability distributions of resistance and load effects established during design or assessment stages. These distributions are typically treated as static and do not evolve as the structure ages or as additional field evidence becomes available [20]. Sensor-informed reliability analysis overcomes this limitation by incorporating measured structural responses, including strain, modal properties, deflection histories, weigh-in-motion measurements, and environmental conditions, to update parameters and refine reliability estimates based on the actual bridge condition [21,22].

The principal mechanism enabling this transition is Bayesian updating, which combines prior knowledge of structural parameters with sensor observations through a likelihood function to yield posterior distributions that reflect measured structural conditions [112,113].

### 3.1. Bayesian Updating of Reliability Models

In Bayesian reliability analysis, prior probability distributions representing initial knowledge about structural resistance, load effects, or deterioration parameters are updated as sensor data becomes available. Each new observation is encoded in a likelihood function that expresses how probable the observed data are under different values of the uncertain parameters. Combining the likelihood with the prior via Bayes’ theorem yields the posterior distribution, which concentrates probability on parameter values consistent with both prior knowledge and field evidence [114,115,116]:

(1) $$\;P\left(\theta \;|\;D\right)=\;\frac{P\left(D\;|\;\theta \right)\cdot P\left(\theta \right)}{P\left(D\right)},$$

where $P\left(\theta \;|\;D\right)$ represents the posterior probability distribution of parameters, θ, given observed data D; $P\left(D|\theta \right)$ is the likelihood function; representing the probability of observing data D given parameters θ; $P\left(\theta \right)$ is the prior probability distribution; and $P\left(D\right)$ is the evidence or marginal likelihood, which acts as a normalizing constant. Here θ denotes the vector of uncertain structural parameters, including resistance, load-effect, and deterioration model parameters, whose posterior $P\left(D|\theta \right)$ is the target of inference.

For continuous SHM applications, monitoring data arrives sequentially. Therefore, the posterior obtained from one update cycle becomes the prior distribution for the next cycle, producing a recursive refinement process [22,23]:

(2) $$P\left(\theta \;|D_{1},D_{2},\;\ldots ,D_{n}\right)\;\propto \;P\left(D_{n}\;|\;\theta \right)\cdot \;P\left(\theta \;|\;D_{1},\ldots ,D_{n-1}\right).$$

This recursive structure allows bridge reliability models to continuously incorporate new information, such as daily strain measurements, updated modal properties, or WIM observations without requiring the entire history to be reprocessed. The resulting posterior distributions (obtained from Equations (1) and (2)) can be propagated through structural reliability algorithms to yield updated failure probabilities and reliability indices that reflect measured structural conditions.

Within bridge SHM, Bayesian updating encompasses four related but distinct applications. (i) Bayesian model updating infers uncertain structural parameters, such as stiffness, damping, and deterioration rates, using measured responses including strains, deflections, or modal properties [114,115,116,117]; (ii) Bayesian state estimation tracks time-varying states, such as fatigue crack growth or section loss, using sequential sensor observations and recursive filtering methods [109,110,116]; (iii) Bayesian sensor fusion combines heterogeneous measurements from strain gauges, WIM systems, temperature sensors, and accelerometers within a unified probabilistic framework [66,118], and (iv) reliability updating propagates the resulting posteriors distributions through reliability models to compute updated failure probabilities and reliability indices [119,120,121]. Although these approaches are interconnected, they address different inferential objectives. Updating modal parameters from vibration measurements is fundamentally different from estimating deterioration-state variables or updating failure probabilities. Consequently, the likelihood function differs for each sensor type. For example, strain and deflection measurements are commonly represented using Gaussian likelihood functions [65], modal properties through eigenvalue-based likelihoods derived from operational modal analysis [67,122,123], WIM measurements through extreme-value distributions [64], environmental effects through covariate-based models [27], and deterioration observations through damage-state or time-dependent likelihood formulations [116].

A critical limitation in sensor-informed reliability analysis is parameter identifiability. While monitoring data may significantly reduce uncertainty in observable parameters, important reliability variables such as structural capacity, deterioration rates, boundary conditions, and load models may remain weakly identified [66,111]. Beck and Katafygiotis [124,125] showed that a model class may contain multiple parameter combinations producing identical outputs at observed degrees of freedom, making the posterior multimodal or flat rather than concentrated. Four complementary diagnostics are available before inference. Firstly, sensitivity analysis identifies parameters to which model outputs are insensitive. Secondly, sloppiness analysis examines the eigenvalue spectrum of the FIM, with small eigenvalues indicating poorly constrained parameters [69]. Thirdly, profile likelihood constructs a one-dimensional likelihood profile for each parameter; a flat profile diagnoses practical non-identifiability. Fourthly, observability analysis determines whether the full state vector can be reconstructed from available measurements, a necessary condition for reliable Bayesian state estimation. These diagnostics confirm that the sensor configuration and model class support meaningful posterior concentration before inference is applied.

Model discrepancy is a related limitation, defined as the systematic gap between the assumed structural model and the actual bridge behavior. In vibration-based model updating, the dominant source of uncertainty is typically model discrepancy combined with identification uncertainty rather than sensor noise [31]. When model discrepancy is unaccounted for, Bayesian updating compensates by absorbing the discrepancy into the structural parameters, producing biased posteriors and overconfident reliability predictions [126].

The formal framework for treating model discrepancy was established by Kennedy and O’Hagan [106], who decomposed the model output into a true physical response term and an additive discrepancy term, both estimated jointly in a Bayesian calibration. Brynjarsdóttir and O’Hagan [126] demonstrated explicitly that ignoring model discrepancy leads to biased and overconfident parameter estimates even when the model is fitted to dense data. Simoen, De Roeck, and Lombaert [31] provide the canonical review of all uncertainty sources in vibration-based finite element model updating for damage assessment, distinguishing measurement noise, model parameter uncertainty, model discrepancy, and identification uncertainty in a unified Bayesian framework. Beck and Katafygiotis [125] and Beck and Au [127] established the computational foundations of Bayesian model updating in structural dynamics that underpin all subsequent sensor-informed reliability work.

For sensor-informed bridge reliability, model discrepancy propagates directly into the posterior distributions of load-effect and resistance parameters that govern *β*. The clean-then-infer architecture discussed in Section 2.4 is particularly vulnerable: when filtered sensor residuals are treated as independent Gaussian observations in the likelihood, any systematic model error is absorbed into the structural parameter estimates rather than isolated as a separate discrepancy term. The joint inference approach and the explicit discrepancy modelling of Kennedy and O’Hagan [106] are the principal remedies. 

Despite its theoretical advantages, direct evaluation of the posterior distribution in Equation (1) is generally computationally intractable for bridge applications involving nonlinear structural models, high-dimensional parameter spaces, and heterogeneous sensor data. This requires integration over the entire parameter space, and the resulting posterior distribution rarely has a closed analytical solution. Therefore, computational methods directly influence uncertainty representation, computational efficacy, and the suitability for real-time or offline reliability assessment.

Table 5 organizes all methods reviewed in this section along six consistent axes. The five core methods in Section 3.1.1 directly approximate *P*(*θ*|*D*) for general high-dimensional and nonlinear problems; the four supporting methods in Section 3.1.2 reduce forward-model cost, exploit analytical structures for special cases, or couple inference with reliability algorithms.

#### 3.1.1. Core Posterior Inference Methods

Core posterior inference methods are computational techniques that estimate the posterior distribution of uncertain structural parameters by integrating sensor data with probabilistic structural models. In Bayesian bridge reliability analysis, evaluating the posterior distribution is rarely straightforward because practical problems involving high-dimensional parameter spaces, nonlinear structural responses, and complex likelihood functions that resist analytical treatment. Consequently, numerical inference methods are required to approximate the posterior distribution and enable sensor-informed reliability assessment.

As shown in Table 5, the five methods reviewed in this section target static structural parameters θ with explicit or implicit likelihoods under batch or sequential data modes. MCMC, SMC, variational inference, and ABC are broad inference families rather than implementations. BayesFlow is an amortized neural posterior estimation framework within the broader neural SBI family, which also includes neural likelihood, and ratio estimation approaches not reviewed here. These five methods represent a chosen scope for bridge reliability applications rather than an exhaustive classification and are compared in Table 6 [113,117,128]. Established methods including MCMC and SMC have been successfully applied to sensor-informed bridge reliability analysis [27,65,67,108,109,110,130,149]. In contrast, emerging methods, such as variational inference, ABC, and neural SBI, have shown promise but have not yet been widely demonstrated for code-relevant bridge reliability index estimation [131,132,133,135,136,138].

**Figure 3:**
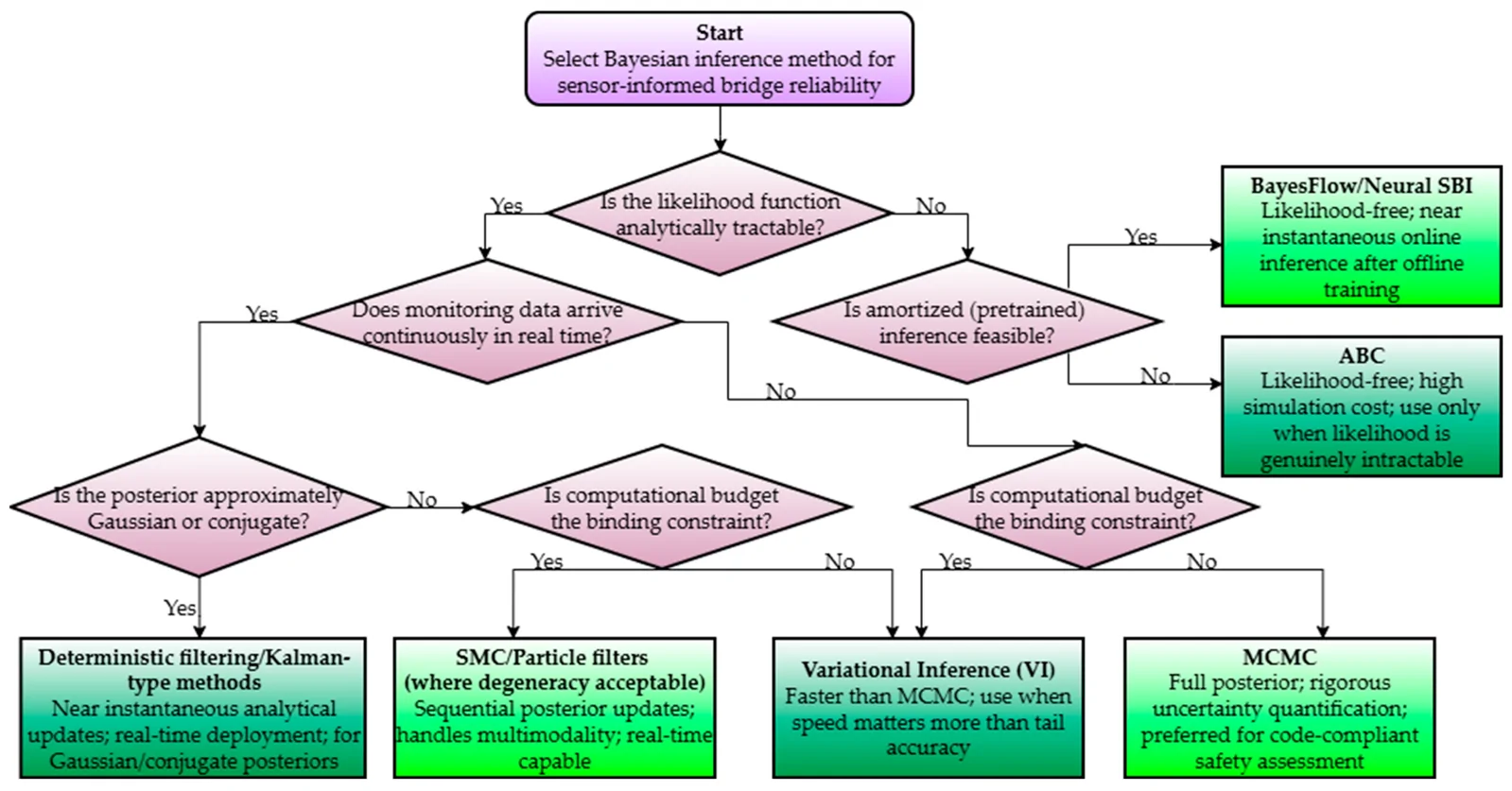
Decision framework for selecting a computational Bayesian inference method. Axes correspond to four of the six taxonomy axes in Table 5. Note that a tractable likelihood does not exclude variational inference; the framework reflects practical deployment priorities. Created by the authors.

The following subsections review each method with respect to its theoretical basis, computational properties, bridge SHM applications, advantages, limitations, and suitability for sensor-informed probabilistic bridge reliability analysis. Their comparative characteristics are summarized in Table 6 and Figure 3.

##### Sampling-Based Methods

Sampling-based methods are the most widely adopted computational approaches for Bayesian inference in bridge reliability analysis. Their popularity stems from both a well-established theoretical foundation [113,117,128] and extensive validation using real bridge monitoring data [65,67,108,149]. Unlike approximate inference techniques, sampling-based methods estimate the full posterior distribution of uncertain structural parameters without imposing restrictive assumptions on its shape. This capability is particularly valuable for bridge applications, where nonlinear structural behavior, heterogeneous sensor data, and multimodal probability distributions are common. Among these methods, MCMC remains the standard approach for offline Bayesian inference. Algorithms such as Metropolis–Hastings and Gibbs sampling generate correlated samples from the posterior distribution after a burn-in period, enabling estimation of structural parameters, failure probabilities, and reliability indices. MCMC is particularly suitable when monitoring data is processed in batches and the computational cost of repeated sampling is acceptable.

A representative bridge application was reported by Ni and Chen [65], who analyzed one year of strain-monitoring data from the Tsing Ma Suspension Bridge in Hong Kong. Using a parametric Bayesian mixture model estimated by Gibbs sampling, they derived posterior distributions of load-effect parameters and subsequently updated component-level reliability indices using FORM. Because strain measurements provide direct observations of structural load effects, the approach enabled estimation of the load-effect probability density function without requiring structural model inversion [65]. However, reliable application depended on rigorous preprocessing, including thermal compensation and drift correction, since systematic measurement errors directly bias the estimated load-effect PDF and the resulting *β* [77,79]. MCMC has also been applied to long-term deterioration assessment. Tonelli et al. [149] combined MCMC-based parameter estimation with importance sampling to update creep and shrinkage parameters of a prestressed concrete bridge using long-term deflection measurements. Their results showed that monitoring-informed Bayesian updating substantially reduced parameter uncertainty and produced more credible estimates of failure probability.

Standard single-chain MCMC methods become less efficient when posterior distributions are highly multimodal or involve many uncertain parameters, as is often the case in finite-element model updating. Transitional Markov Chain Monte Carlo (TMCMC), introduced by Ching and Chen [129], addresses this limitation by introducing a sequence of intermediate distributions that gradually transform the prior into the target posterior. Besides improving sampling efficiency, TMCMC simultaneously estimates the marginal likelihood, enabling probabilistic comparison of competing structural models.

Asadollahi et al. [67] demonstrated the advantages of TMCMC using one year of wireless accelerometer data collected from the Jindo cable-stayed bridge in South Korea. The method successfully updated a high-dimensional finite-element model while simultaneously evaluating competing model classes through marginal likelihood estimation. Accelerometers offer significant practical advantages because they enable non-intrusive, network-based monitoring of global structural dynamics. However, reliability assessment based on modal parameters requires structural model inversion, introducing additional uncertainty compared with direct load-effect measurements obtained from strain monitoring [66,67].

When multiple sensing modalities are available, hierarchical Bayesian models provide a natural extension of conventional MCMC by explicitly accounting for variability among different data sources. Rather than pooling heterogeneous measurements directly, hierarchical formulations estimate shared structural parameters while preserving sensor-specific uncertainty. Luo et al. [108] demonstrated this concept by integrating video, WIM, and vibration measurements from a laboratory three-span bridge to obtain joint posterior estimates of stiffness parameters with improved uncertainty characterization.

The fundamental limitation of MCMC methods is that they are designed for static datasets and become computationally expensive when new monitoring data are continuously acquired. Repeating a complete MCMC analysis after every monitoring update is generally impractical for long-term bridge SHM.

Sequential Monte Carlo (SMC) methods, commonly known as particle filters, overcome this limitation by representing the posterior distribution with a population of weighted particles that are updated incrementally as new measurements become available. This recursive formulation naturally supports online Bayesian updating, accommodates multimodal posterior distributions, and is readily parallelized, making SMC particularly attractive for real-time bridge reliability assessment.

Fan, Liu and Lü [109] first demonstrated the feasibility of Gaussian particle filter for online reliability prediction using monitored extreme stress data from bridge members. Fan and Liu [110] subsequently refined the approach by combining dynamic linear models with a Gaussian mixed particle filter, achieving greater prediction accuracy and stability during long-term monitoring. More recently, Qu and Sun [130] coupled an improved particle filter with a First-Order Second Moment (FOSM) reliability method, enabling dynamic estimation of reliability indices at critical locations of an in-service bridge. The broader applicability of sequential Bayesian inference was further illustrated by Ali et al. [150], who applied an SMC-based particle filter with Micro-Electro-Mechanical Systems (MEMS) accelerometer measurements from the Jinnah Bridge in Pakistan to recursively estimate structural health states and predict RSL through multi-step probabilistic forecasting.

##### Optimization-Based Approximate Methods 

While sampling-based methods offer rigorous uncertainty quantification, their computational demands grow substantially with model complexity and parameter dimensionality. This limitation becomes particularly significant in large-scale bridge SHM applications, where repeated likelihood evaluations using high-fidelity finite element models can be computationally prohibitive. Optimization-based approximate methods address this bottleneck, by replacing computationally intensive sampling with optimization procedures that approximate the posterior distribution while substantially reducing computational cost [132,152].

The most widely used optimization-based approach is Variational Inference (VI). Rather than generating samples from the posterior distribution, VI formulates Bayesian inference as an optimization problem. A tractable family of probability distributions is selected, and the member that most closely approximates the true posterior is identified by minimizing the Kullback–Leibler (KL) divergence or, equivalently, maximizing the Evidence Lower Bound (ELBO) [131]. This deterministic formulation enables efficient gradient-based optimization, scales well to high-dimensional problems, and is readily integrated with modern machine learning framework [131,133].

Compared to MCMC, VI offers substantially faster inference and is therefore attractive for large monitoring datasets and repeated Bayesian updating. However, the standard reverse-KL formulation exhibits a well-known mode-seeking behavior, often underestimating posterior variance and providing overconfident uncertainty estimates, particularly in the tails of the distribution [131,133]. Consequently, VI is generally more suitable for computationally demanding monitoring applications than for reliability assessments where accurate estimation of low-probability failure events is critical.

Recent developments have significantly expanded the capabilities of VI. Advances in stochastic gradient optimization, non-conjugate VI, structured variational approximations, and amortized inference have improved both scalability and approximation accuracy, making VI applicable to increasingly complex structural models [133].

Several studies have demonstrated the potential of VI for structural model updating and SHM applications. Ni et al. [132] combined Gaussian process surrogate modelling with Gaussian mixture VI to efficiently estimate posterior distributions for structural model updating and damage identification. The methodology was validated using an eight-story shear-type building, a simply supported beam, and a laboratory steel frame structure, demonstrating that surrogate-assisted VI can substantially reduce the computational burden associated with expensive likelihood evaluations. Jia et al. [134] further developed a hierarchical Bayesian model updating framework based on VI, deriving analytical posterior approximations both model parameters and hyperparameters, thereby eliminating the need for computationally intensive MCMC sampling. Although these studies focus primarily on structural dynamics rather than bridge reliability, the underlying frameworks are directly applicable to bridge Finite Element (FE) model updating.

Bridge-specific applications remain relatively limited. Ma et al. [151] applied sparse variational GPR to long-term monitoring of temperature and bearing displacement, developing a probabilistic prediction and early-warning framework capable of handling large SHM datasets that would be computationally infeasible for conventional Gaussian process models. While this work demonstrates the scalability of variational methods for bridge monitoring, it focuses on condition prediction rather than direct computation of reliability indices. Aloisio et al. [153] demonstrated Bayesian estimation of elastic modulus from dynamic identification of concrete box girders on the A24 motorway in Italy, providing a bridge-specific application of posterior parameter estimation for maintenance prioritization.

Compared with the sampling-based approaches discussed in Section Sampling-Based Methods, optimization-based approximate methods serve a complementary role in sensor-informed bridge reliability assessment. MCMC and SMC remain preferable when accurate full-posterior uncertainty quantification is required, particularly for offline analyses where reliability estimates must satisfy rigorous safety requirements. In contrast, variational inference becomes attractive when monitoring data streams are large, fast repeated batch inference is required rather than genuine sequential online updating, or repeated probabilistic inference must be performed across multiple structural scenarios. The bridge-specific VI application identified here [151] focuses on condition monitoring and prediction rather than direct computation of formal reliability indices, indicating that further research is needed before VI can be considered a mature alternative for reliability-based bridge decision-making [132,134].

##### Likelihood-Free (Simulation-Based) Methods 

Both sampling-based and optimization-based methods require an explicit likelihood function to perform Bayesian inference, Pseudo-marginal MCMC and synthetic likelihood provide intermediate solutions when the likelihood is expensive but not fully intractable. While this requirement is satisfied for many bridge reliability problems, it becomes restrictive when the likelihood is analytically intractable, computationally expensive to evaluate, or impossible to formulate because of highly nonlinear finite-element models or black-box structural simulators [137]. Likelihood-free, or simulation-based, inference methods address this constraint by replacing likelihood evaluation with repeated forward simulations. Candidate parameter values are used to generate synthetic structural responses, which are then compared with observed sensor data to approximate the posterior distribution.

The most established likelihood-free approach is Approximate Bayesian Computation (ABC). Rather than evaluating the likelihood directly, ABC samples candidate parameter values from the prior, simulates structural responses, and accepts parameter sets whose simulated outputs closely match the observed data according to predefined summary statistics and a tolerance threshold [135,136]. This strategy enables Bayesian inference without requiring an analytical likelihood function, making ABC particularly attractive for complex nonlinear structural models and computationally intensive FE simulations.

Despite this flexibility, ABC has several practical limitations. Its computational efficiency depends strongly on the choice of summary statistics, distance metrics, and acceptance tolerance, while poor selection of summary statistics can result in substantial information loss, particularly for high-dimensional SHM datasets such as distributed strain measurements or modal response histories [135,136]. Consequently, ABC generally requires a large number of forward simulations and is considerably more computationally demanding than likelihood-based Bayesian methods when an explicit likelihood is available.

The applicability of ABC to structural model updating was demonstrated by Monchetti et al. [154], who compared conventional Bayesian model updating and ABC for identifying uncertain parameters of a historic masonry tower using ambient vibration measurements. Although the study did not involve a bridge, it showed that ABC produced posterior parameter estimates comparable to likelihood-based Bayesian updating while remaining applicable when the likelihood became computationally intractable. These characteristics make ABC potentially valuable for bridge finite-element models involving numerous uncertain material and geometric parameters. However, applications to formal bridge reliability index computation and failure probability estimation remain scarce.

More recently, neural Simulation-Based Inference (SBI) has emerged as a promising alternative to conventional ABC. Frameworks such as BayesFlow [137,138] address the two most significant limitations of ABC. First, they eliminate the need for user-defined summary statistics by jointly training a summary network and an invertible neural network on structural model simulations, enabling the full probabilistic mapping from sensor observations to posterior distributions to be learned directly from data [137]. Second, the amortized training approach means posterior inference on new monitoring datasets is performed almost instantaneously without rerunning forward models or evaluating likelihoods, making continuous deployment viable in a way conventional ABC is not [136,154]. This makes neural SBI particularly attractive for continuous SHM applications where repeated Bayesian updating is required.

Martínez and Lathourakis [138] demonstrated the potential of BayesFlow for bridge monitoring by applying it to damage detection using simulated influence-line measurements, cable axial forces, and displacement responses under moving traffic loads. The trained network accurately estimated both damage location and severity while producing posterior distributions comparable to those obtained through conventional Bayesian inference. Although this work illustrates the computational efficiency and scalability of neural SBI for bridge SHM, its application has so far been limited to damage detection rather than direct computation of reliability indices or failure probabilities.

Regardless of the inference method employed, the quality of posterior estimates ultimately depends on the quality of the input monitoring data. Sensor imperfections reviewed in Section 2.3 can each corrupt the likelihood function *L*(*D*|*θ*) in distinct ways: a corrupted likelihood produces a biased posterior *P*(*θ*|*D*); a biased posterior propagates into erroneous reliability index *β* and RSL estimates; and erroneous *β* and RSL propagate into suboptimal maintenance decisions. This propagation chain, illustrated for drift and bias in Table 3 and formalized in the observation model $D\left(t\right)={x\left(t\right)+\delta }_{d}\left(t\right)+\delta_{b}+\varepsilon \left(t\right)$, applies analogously to all imperfection types reviewed in Section 2.3. Therefore, the preprocessing strategies discussed in Section 2 remain a fundamental prerequisite for reliable Bayesian inference and code-compliant bridge reliability assessment [22,79].

#### 3.1.2. Supporting Inference Methods

The core posterior inference methods reviewed in Section 3.1.1 provide the foundation for Bayesian updating in sensor-informed bridge reliability analysis. Their practical application to real bridge structures, however, is often constrained by recurring computational challenges. High-fidelity finite element and stochastic deterioration models are computationally expensive to evaluate, monitoring data are acquired continuously over long service lives, and decision-oriented quantities such as reliability indices, failure probabilities, and value-of-information metrics must be updated repeatedly under multiple maintenance and deterioration scenarios.

Supporting inference methods complement the core posterior inference approaches by improving computational efficiency, exploiting approximate analytical structures, or facilitating tighter coupling between Bayesian updating and structural reliability analysis. Rather than replacing the core inference methods, they enhance their practical applicability for large-scale and long-term bridge SHM problems.

The four supporting methods reviewed here reduce forward-model cost, exploit analytical structures for special cases, or couple inference with reliability algorithms, rather than directly targeting the general posterior. Their comparative characteristics are summarized in Table 7.

##### Surrogate/Metamodel-Based Methods

Surrogate and metamodel-based methods are the most widely adopted supporting inference techniques in sensor-informed bridge reliability analysis. Their primary objective is to replace computationally expensive forward structural models with computationally efficient approximations, thereby substantially reducing the cost of repeated likelihood evaluations during Bayesian updating. By constructing a surrogate model offline, posterior inference can be performed using inexpensive model evaluations rather than repeated finite-element (FE) analyses, making Bayesian reliability assessment practical for large-scale bridge systems and long-term SHM applications. The principal limitation is that approximation errors in the surrogate model may propagate into the posterior distribution and, consequently, into the estimated reliability indices.

A variety of surrogate architectures have been applied to bridge reliability problems, including neural network surrogates, Gaussian process models, response surface methods, polynomial chaos expansion, and multi-fidelity FE surrogates [139,140,141,142,155,156]. The selection of a surrogate depends on the complexity of the structural model, the available computational resources, and the required balance between prediction accuracy and computational efficiency.

Deep learning-based surrogates have demonstrated particular promise for computationally intensive Bayesian updating. Torzoni et al. [139] developed a multi-fidelity deep neural network surrogate that combines high-fidelity and reduced-order FE simulations to relate structural damage and operational parameters to measured sensor responses. Embedded within an MCMC framework, the surrogate enabled efficient posterior updating of the structural state of a portal-frame railway bridge under operational variability and measurement noise. Similarly, He et al. [155] adopted a back-propagation neural network surrogate, enhanced using an artificial bee colony optimization algorithm, to compensate for environmental effects in bridge frequency measurements before propagating the updated structural parameters through a dynamic Bayesian network for time-dependent reliability assessment.

**Table 7:** Comparison of supporting inference methods for sensor-informed bridge reliability assessment. Bridge applications: demonstrated only; Best Suited For: prospective suitability.

Supporting Methods	Computational Cost	Role in Reliability Pipeline	Real-time Applicability	Required Assumptions	Primary Limitation	Best Suited for	Bridge Applications
Surrogate/Metamodel-Based Methods	High offline training: low online; surrogate calls are far cheaper than direct FE evaluation	Replaces expensive FE model with trained surrogate used within MCMC or SMC to reduce per-likelihood evaluation cost	Yes	No assumptions on posterior form; accuracy depends on training coverage	Approximation error propagates into posterior and reliability; unreliable beyond training domain	High-fidelity bridge models where direct FE is too costly for repeated updates; continuous monitoring deployments	[139,140,141,142,155]
Deterministic Numerical Integration & Filtering	Very low; algebraic operations only; no FE evaluations or sampling chains	Exploits conjugate or Gaussian structure for closed form or recursive analytical updates without sampling	Yes	Requires state-space structure; Kalman Filter needs linear–Gaussian transition and observation models	Unsuitable for nonlinear or multimodal bridge deterioration models	Low-dimensional, near-Gaussian problems requiring near-instantaneous updates from streaming sensor data	[25,143]
Asymptotic Large-Sample Approximations	Low; MAP optimization plus Hessian; avoids iterative sampling chains	Approximates posterior near MAP via Laplace expansion for closed-form posterior samples and marginal likelihoods	Partial	Requires sufficiently informative data and approximately Gaussian, unimodal posterior distributions	Tail approximation unreliable for low failure probabilities; degrades for non-Gaussian posteriors	Decision-oriented lifecycle studies requiring repeated reliability evaluations under many monitoring or deterioration scenarios	[144,145]
Hybrid Bayesian–Reliability Frameworks	High; full posterior sampling plus reliability computation; requires surrogate acceleration or parallelization	Couples Bayesian posterior updating directly with FORM, SORM, importance sampling, or subset simulation so reliability indices are native outputs	No: offline batch updates only	None, on posterior form inherits assumptions of the chosen reliability algorithm	High computational cost; depends on surrogate acceleration or parallelization for large bridge models	Applications requiring tight coupling between posterior uncertainty and code-compliant safety metrics; formal reliability index as direct output	[24,65,115,146,157]

Physics-based surrogate models have also been successfully integrated with Bayesian inference. Chen et al. [140] combined an intrusive homotopy stochastic finite-element surrogate with delayed-rejection adaptive Metropolis sampling to update the modal properties of a cable-stayed bridge from uncertain vibration measurements. Compared with second-order perturbation, response-surface, and Kriging-based approaches, the homotopy surrogate produced more accurate posterior estimates while maintaining computational efficiency.

Response-surface and polynomial-based surrogates offer a complementary strategy for reducing computational demand. García-Macías and Ubertini [141] combine adaptive sparse polynomial chaos expansion with Kriging within an adaptive MCMC framework to achieve Bayesian damage identification from continuous ambient vibration measurements. Dai et al. [142] employed multi-output support vector regression together with response-surface techniques to calibrate a detailed FE model of the Yingzhou steel arch bridge, enabling computationally efficient fatigue assessment using long-term SHM data. Earlier work by Worden and Cross [156] demonstrated the effectiveness of Treed Gaussian Process models for separating environmental and operational variability from SHM and Bayesian model updating.

##### Deterministic Numerical Integration and Filtering Methods

Deterministic numerical integration and filtering methods, of which Kalman-type filters and their nonlinear extensions are canonical implementation, provide an efficient alternative to sampling-based Bayesian inference when the probabilistic model is low to moderate-dimensional, approximately Gaussian, or possesses conjugate analytical structure. Rather than approximating the posterior through stochastic sampling, these methods exploit analytic or semi-analytic solutions, numerical quadrature, or recursive state-space estimation to compute posterior moments and predictive distributions. Their low computational cost and recursive formulation make them particularly well suited to real-time bridge SHM applications, where sensor measurements are acquired continuously and posterior estimates must be updated as new data become available.

Analytical Bayesian updating is possible when conjugate prior–likelihood relationships exist. Ni et al. [25] demonstrated this using long-term monitoring data from the Ting Kau cable-stayed bridge by developing a Bayesian linear regression model relating temperature to expansion-joint displacement. Owing to the conjugate formulation, the posterior distribution of the regression coefficients was obtained analytically, and a reliability-based anomaly index was computed directly from the posterior predictive distribution without requiring MCMC sampling. This enabled near-instantaneous condition assessment and damage detection from streaming sensor measurements.

Recursive state-space filtering extends deterministic Bayesian inference to time-varying bridge monitoring problems. After preprocessing bridge frequency measurements using a neural-network surrogate, He et al. [155] embedded the updated bridge parameter distributions within a dynamic Bayesian network recursively propagated the posterior through a bridge reliability model to obtain time-dependent deflection reliability indices. This framework illustrates how recursive Bayesian updating can continuously transform monitoring sensor data into reliability metrics suitable for long-term bridge monitoring.

Deterministic recursive updating has also been applied to time-dependent reliability prediction under cyclic loading. Qu et al. [143] proposed a Gaussian mixture model-based Bayesian cyclical dynamic linear model to describe extreme stress time series obtained from bridge SHM systems. Posterior moments were updated recursively from monitoring data and combined with the FOSM method to predict the time-varying reliability of an in-service bridge. By explicitly accounting for seasonal and cyclic variations in monitored stresses, the framework provided more realistic reliability predictions for long-term bridge operation.

##### Asymptotic Large-Sample Approximations

Asymptotic large-sample approximation methods exploit the tendency of posterior distributions to converge toward a multivariate Gaussian distribution as the amount of monitoring data increases under standard regularity conditions. Instead of performing computationally intensive sampling, these methods approximate the posterior locally around the maximum a posteriori (MAP) estimate, with covariance given by the inverse Hessian of the negative log-posterior. The resulting Laplace approximation provides closed-form expressions of posterior distributions and marginal likelihoods, enabling efficient uncertainty propagation and rapid reliability evaluation.

The principal advantage of asymptotic methods in bridge reliability analysis is computational efficiency. Because the posterior is represented analytically near its mode, reliability indices and failure probabilities can be evaluated repeatedly under different monitoring, deterioration, or maintenance scenarios at a fraction of the computational cost required by sampling-based methods. Their main limitation is that approximation accuracy degrades when posterior distributions are highly non-Gaussian, multimodal, or strongly skewed, where the Laplace approximation may inadequately represent the posterior tails that govern structural reliability.

He et al. [145] demonstrated the application of Laplace approximations combined with FORM and inverse FORM to construct an efficient Bayesian reliability workflow for bridge structure, explicitly addressing the challenges posed by multimodal posterior distributions and limit-state functions. Tong et al. [144] further integrated a neural network surrogate with Bayesian inference and asymptotic approximation to predict the long-term reliability of a prestressed concrete bridge under monitoring-informed uncertainties, illustrating how asymptotic inference can support efficient reliability updating for computationally demanding bridge SHM applications.

##### Hybrid Bayesian–Reliability

Hybrid Bayesian–reliability frameworks describe a coupling architecture between Bayesian inference and structural reliability analysis rather than an inference method in the same sense as MCMC or VI; they directly couple Bayesian parameter or state updating with classical reliability algorithms such as FORM, SORM, importance sampling, or subset simulation. Unlike conventional workflows in which Bayesian updating and reliability assessment are performed separately, hybrid approaches propagate posterior parameter distributions directly into reliability calculations, enabling continuous estimation of reliability indices, failure probabilities, RSL, and maintenance decision metrics from SHM data, although high-fidelity implementations are typically offline due to computational cost.

The principal advantage of hybrid frameworks is their ability to convert monitoring data into actionable reliability information without intermediate post-processing. This direct integration makes them particularly attractive for long-term bridge management, where reliability estimates must be updated repeatedly as new monitoring data become available. Their main limitation is computational cost, especially for high-dimensional bridge models, often requiring surrogate modelling, parallel computing, or efficient inference described in the preceding subsections.

Straub [147] established reliability updating with equality and inequality information, and Straub and Papaioannou [148] introduced the BUS approach, which reformulates Bayesian updating as a structural reliability problem. Several representative studies demonstrate the effectiveness of this integrated framework. Ni and Chen [65] and Chen and Ni [157] combined Bayesian mixture with FORM to evaluate the conditional reliability of the Tsing Ma Bridge using long-term strain monitoring data. Bayesian updating continuously refined the multimodal load-effect distributions, while FORM translated the updated posterior into reliability indices that evolved as additional monitoring data became available. Jerez et al. [115] exploited the mathematical equivalence between Bayesian updating and structural reliability to implement subset simulation for simultaneous Bayesian model updating and reliability estimation on a nonlinear three-dimensional bridge model. Simon et al. [146] extended this framework by parallelizing the Bayesian Updating with Structural reliability methods (BUS) algorithm using subset simulation and applying it to the Maintalbrücke Gemünden railway bridge, demonstrating that computationally demanding Bayesian updating for large SHM-oriented models can be performed efficiently through parallel computing. At the decision-making level, Kamariotis et al. [24] incorporated Bayesian model updating of modal properties into a lifecycle reliability framework, combining sequential reliability updating with pre-posterior Bayesian decision analysis to quantify the value of vibration-based SHM information for bridge maintenance planning. Figueiredo and Brownjohn [27] reviewed three decades of statistical pattern recognition for bridge SHM, highlighting Bayesian learning and reliability-based decision support as key components of modern monitoring frameworks.

### 3.2. Sensor-Informed Reliability Index

The posterior distributions obtained from the methods of Section 3.1 are integrated with structural reliability models to compute updated failure probabilities and reliability indices. In conventional reliability analysis, structural safety is evaluated through a limit-state function defined in terms of structural resistance *R* and applied load *S*:

(3) $$G\left(R,S\right)=R-S,$$

where R represents structural resistance and *S* denote the applied load which can be estimated directly or indirectly from sensor monitoring data. Failure occurs when the limit-state function becomes zero or negative (G(X)≤0), whereas G(X)>0 represents the safe domain. The corresponding failure probability $\left(P_{f}\right)$ is therefore expressed as:

(4) $$P_{f}=P\left(G\left(X\right)\leq 0\right)=\int_{G\left(X\right)\leq 0}f_{X}\left(x\right)dx,\;$$

where $X=\left(R,\;S,\ldots \right)$ is the vector of basic random variables, $f_{X}\left(x\right)$ is their joint probability density function, the integration is performed over the failure domain. In general, this integral does not have a closed-form solution and is evaluated using numerical reliability methods such as FORM, SORM, Monte Carlo simulation, or subset simulation [10,15,16,158].

In a sensor-informed reliability assessment, *R* and *S* are no longer treated as fixed design-stage quantities; instead, they are represented by posterior-updated distributions derived from field measurement [119]. The reliability index at time t, denoted as *β(t)*, represents the distance between the mean performance point and the failure boundary in the standard normal space. Under the FOSM approximation, where independent normal *R* and *S* and correlated or non-Gaussian cases require FORM with Nataf or Rosenblatt transformation, SORM, or simulation, the reliability index is expressed as [8,9,10,111]:

(5) $$\beta \left(t\right)=\;\frac{\mu_{R}-\mu_{s}}{\sqrt{\left(\sigma_{R}^{2}\;+\;\sigma_{S}^{2}\right)}},$$

under the standard normal assumption, *β*(*t*) is related to the failure probability $P_{f}$ through the inverse standard normal CDF $\Phi^{-1}$ as:

(6) $$\beta \left(t\right)=\;\Phi^{-1}\left(1-\;P_{f}\right),$$

where $\mu_{R}$ and $\mu_{S}$ represent the mean resistance and load-effect values, respectively, and $\sigma_{R}$ and $\sigma_{S}$ are their corresponding standard deviations. Equation (5) is the Cornell second-moment reliability index. This index is not invariant to the algebraic form of the limit-state function [8]. Hasofer and Lind [8] handle the limitation by reformulating *β* as the minimum distance from the origin to the limit-state surface in standardized normal space, forming the basis of modern FORM. For nonlinear limit states, correlated variables, or non-Gaussian distributions, more advanced methods including FORM, SORM, and simulation-based approaches are required [10,15,16]. From the posterior distribution *P*(*θ*|*D*), three reliability quantities can be computed and must not be conflated. Firstly, β(θ) conditional on fixed parameter values. Secondly, the posterior predictive $\beta_{pred.}=\Phi^{-1}\left(1-\;\int P_{f}(failure|\theta )p(\theta |D)d\theta \right)$, which accounts for both aleatory and epistemic uncertainty and is most relevant to code-compliant safety assessment, subject to the applicable limit state, reference period, consequence class, component or system level, and normative framework. Thirdly, the posterior mean *E*[*β*(*θ*)|*D*], which differs numerically from $\beta_{pred.}$ due to the nonlinearity of $\Phi^{-1}$ [147,148,159]. In the authors’ own notation, *β* refers to the posterior predictive quantity; cited historical studies retain the reliability index definitions. Aleatory variability is captured in the conditional distributions of *R* and *S* given θ; epistemic uncertainty is captured by *P*(θ|*D*).

The reliability index *β* from Equations (5) and (6) is an instantaneous measure at time t. A complete time-variant reliability assessment requires computing the probability that the structure remains safe throughout a reference period, which differs fundamentally from $P_{f}(t)$. The cumulative failure probability $P_{f}(0,t)$ accounts for all load excursions up to time t and exceeds $P_{f}(t)$ over any finite reference period; Zhu and Frangopol [160] demonstrated this distinction for bridge risk assessment. Rice [161] established the outcrossing-rate framework, relating $P_{f}(0,t)$ to the mean rate at which the limit-state process crosses from safe to failure domain. The PHI2 method of Andrieu-Renaud et al. [162] computes this outcrossing rate using FORM, converting time-variant reliability into a two- component parallel system problem compatible with the sensor-informed updating framework. The Poisson square-wave model represents live loads as rectangular pulses with Poisson-distributed arrival times, while the Ferry Borges–Castanheta model discretizes load processes into blocks of equal duration [163]; both directly determine the outcrossing rate and cumulative failure probability. Sensor-informed Bayesian updating reduces uncertainty in resistance and load parameters, narrowing the distribution of *β*(*t*) and extending the predicted RSL.

Within a sensor-informed framework, reliability indices can be continuously updated as new monitoring information becomes available. The updated $\beta \left(t\right)$ can be computed over moving time windows or sequentially updated posterior distributions, enabling the reliability assessment to reflect the evolving structural condition rather than relying solely on initial design assumptions [111,119]. When the updated reliability index falls below a predefined target reliability level $\beta_{target}$, the results can support automated warning systems, inspection prioritization, or maintenance decision-making for in-service bridges [51,111]. Table 8 summarizes the key features of the reviewed case studies. The following sub-sections review the implementation of this sensor-to-reliability framework across different bridge types, sensor configurations, and structural limit states.

#### 3.2.1. Strain and Stress Sensor Applications

The earliest demonstrations of sensor-informed reliability assessment relied on long-term strain measurements to construct the load-effect distribution *S(t)*, replacing assumed traffic load models with bridge-specific field observations. Frangopol et al. [119] established a foundation for this approach on the Lehigh River Bridge SR-33 in Pennsylvania, where monitored live-load effects were used to update the load-effect probability density function and compute a FORM-based reliability index *β*. A key outcome of this study was that the monitoring-informed reliability estimates differed substantially from design-stage predictions demonstrating that field measurements can modify safety assessments rather than simply reduce uncertainty. The companion papers by the same authors [22,120] generalized this concept into a framework for incorporating monitored extreme events into performance prediction and Bayesian reliability updating, establishing the basis for later sensor-informed reliability applications.

Subsequent work demonstrated that the strain-to-reliability framework is applicable across bridge types, loading conditions, and failure modes. Ni et al. [64] extended this approach to fatigue reliability assessment on the Tsing Ma Suspension Bridge in Hong Kong by extracting hot-spot stress range probability distributions from long-term strain monitoring data and combining them with a probabilistic Miner’s damage accumulation model. Because the stress-range PDF was derived from measured structural response, the resulting fatigue reliability index reflected the actual mixed highway and railway loading environment rather than a generic design spectrum. Similarly, Li et al. [121] demonstrated multi-hazard reliability assessment on a long-span cable-stayed bridge by integrating monitored vehicle loads, wind effects, and environmental measurements to establish component-level reliability indices for the girder, cables, and towers. Catbas et al. [118] further showed that integrating structural response measurements with environmental monitoring data, including temperature, wind, and traffic information, provides a more comprehensive representation of operational variability and load combinations for reliability assessment.

Beyond wind and traffic loading, sensor-informed reliability has been extended to seismic, scour, and flood hazards. Ghosh and Padgett [166] showed that aging deterioration from chloride-induced corrosion significantly modifies seismic fragility curves, demonstrating that static fragility models are insufficient for aging bridges. Rapid post-earthquake damage assessment uses updated model parameters from pre-event SHM to reduce uncertainty in post-event response simulation; Ghahari et al. [165] demonstrated this through sequential Bayesian model updating with recorded seismic measurements, reducing uncertainties in structural and soil–structure interaction parameters for rapid post-earthquake damage diagnosis. For flood-induced scour, which is among the most common external causes of bridge failures worldwide and responsible for 22 bridge failures per year in the United States alone [60]. Maroni et al. [60] demonstrated the first application of Bayesian networks to bridge scour risk assessment, showing how data from a limited number of instrumented bridges can significantly reduce uncertainty in scour depth at unmonitored network locations.

Despite the advantages of sensor-informed reliability assessment across multiple hazard types, heterogeneous sensor data integration introduces additional challenges: each sensor type requires separate correction methods, synchronization across different sampling rates, and independent validation [26,77]. These practical limitations must be weighed against the improved reliability characterization for routine bridge monitoring programs. Collectively, these studies demonstrate that [22,119,120] strain-based reliability updating is not limited to a specific bridge type but provides a general framework for converting measured structural responses into probabilistic safety indicators.

#### 3.2.2. Recursive and Sequential Updating of Reliability Index (*β*)

A further development in sensor-informed reliability assessment is the transition from single-point reliability evaluation toward sequential periodic updating of the reliability index (*β*) as monitoring data accumulate. This capability depends primarily on computationally effective inference approaches that can update posterior distributions without repeatedly performing computationally intensive analyses.

Ni and Chen [65] demonstrated that reliability indices could be updated sequentially as strain monitoring data accumulated on the Tsing Ma Suspension Bridge, demonstrating the feasibility of ongoing reliability assessment without retrospective reanalysis. This approach showed that reliability updating could be performed sequentially as monitoring information accumulated rather than only through retrospective analysis. Similarly, Qu et al. [143] combined a Bayesian cyclical dynamic linear model with FOSM reliability analysis to update reliability indices from extreme stress measurements while explicitly accounting for seasonal variations in structural response.

Particle-based sequential inference methods have further expanded the potential for continuous reliability updating. Fan et al. [109] demonstrated online reliability prediction using a Bayesian dynamic linear model combined with a Gaussian particle filter based on monitored extreme stress data, while Fan and Liu [110] improved long-term prediction stability through a Gaussian mixed particle filter formulation. Qu and Sun [167] subsequently demonstrated that long-term reliability trends could be tracked using an improved Bayesian dynamic regression linear model, highlighting the potential for sustained reliability monitoring over the bridge service periods. Kamariotis et al. [66] incorporated vibration-based monitoring and visual inspection information into a Bayesian filtering framework to update deterioration states and reliability-related parameters.

Together, these studies demonstrate the technical feasibility of recursive reliability updating across multiple sensor types and inference frameworks. However, operational implementation remains dependent on reliable long-term sensing, uncertainty calibration, computational efficiency, and integration with bridge management decision processes. These limitations are particularly relevant when moving from research demonstrations to fully automated reliability-based maintenance systems.

#### 3.2.3. Live Load Monitoring Applications

While strain monitoring provides an indirect measurement of traffic effects through structural response, weigh-in-motion and related load-monitoring technologies provide direct information about the applied load component $S\left(t\right)$. Liu et al. [68] demonstrated this approach on a highway bridge in Wisconsin by deriving load-effect probability density distributions directly from measured structural responses and combining them with resistance PDF derived from material properties and damage assessment. FORM-based reliability indices *β* showed that monitoring duration, measurement uncertainty, and extreme-value distributions significantly influence the resulting safety assessment, confirming that sensor data quality directly affects reliability conclusions.

Kwon and Frangopol [164] extended this approach to fatigue reliability assessment of steel bridges by fitting equivalent stress-range distributions from monitoring data and propagating these through fatigue limit state models. The resulting reliability estimates differed substantially from those obtained using code-based loading assumptions, highlighting the importance of measured operational loading conditions for fatigue-critical structures. However, most existing applications remain focused on steel bridges, where uncertainty is primarily associated with load effects rather than resistance deterioration.

#### 3.2.4. Deflection and Prestress Sensor Applications

For prestressed concrete bridges, the dominant uncertainty often lies in the resistance model rather than the applied loading. Therefore, sensor-informed reliability assessment requires updating resistance-related parameters, including prestress losses, creep, shrinkage, and stiffness degradation, using field measurements. Jia et al. [51] applied Bayesian inference to deflection sensor measurements from prestressed concrete bridge sections to update posterior distributions of creep coefficients, structural rigidity, dead load effects, shrinkage, and prestress parameters. The updated distributions were subsequently used for reliability assessment with reduced epistemic uncertainty compared with design-stage assumptions.

Sarmiento et al. [52] demonstrated the significance of such updating on the Kalix Bridge, a 66-year-old prestressed concrete box-girder bridge in Sweden, by integrating residual prestress measurements and material testing with Bayesian updating. The updated resistance model substantially changed the estimated reliability index compared with the initial assessment based only on design information, illustrating the importance of incorporating measured structural condition into evaluations of aging bridges. Deflection sensors and on-site material testing offer the advantage of capturing integrated creep, shrinkage, and prestress loss effects directly, a global resistance indicator that point strain gauges cannot provide [51,52]. However, long-term displacement monitoring introduces practical challenges related to sensor stability and reference continuity. Replacement or recalibration of displacement sensors resets the baseline and breaks monitoring continuity, introducing a step change that directly affects long-term RSL projections [77,168]. Therefore, maintaining reliable sensor calibration and uncertainty tracking remains essential for resistance-updating approaches.

## 4. Remaining Service Life and Maintenance Performance

The Bayesian inference methods discussed in Section 3.1 provide posterior distributions of uncertain structural parameters, while Section 3.2 demonstrated how these updated uncertainties can be translated into sensor-informed reliability indices *β* and failure probability $P_{f}$. These probabilistic outputs represent the essential link between monitoring data and infrastructure management decisions; however, they are not the final objective of reliability assessment. In practice, reliability information must support two interconnected decisions. The first decision is an immediate operational decision whether the current reliability state has deteriorated sufficiently to require inspection, intervention, or load restriction. The second is a long-term lifecycle decision: how rapidly reliability is expected to decline and when maintenance actions should be implemented to extend RSL. Consequently, sensor-informed reliability assessment provides the foundation for both reliability-based alarm systems and predictive maintenance strategies, where current and future reliability states are explicitly incorporated into decision-making. The selection of inference methods, reliability formulation, and operational decision framework must be jointly considered; Table 6, Table 7 and Table 8 summarize the conditions and limitations of each inference method reviewed in Section 3, and the subsections below address conditions and limitations for each operational approach.

### 4.1. Reliability-Based Alarm Triggering and Immediate Maintenance Decisions

The translation of continuously updated reliability indices into operational alarms requires defining an acceptable reliability threshold $\beta_{target}$ and establishing a decision rule for intervention when the estimated reliability approaches or falls below this limit. This extends beyond a purely probabilistic calculation because the selected threshold must reflect safety requirements, economic constraints, inspection capabilities, and maintenance policies. Ni et al. [25] demonstrated this concept on the Ting Kau cable-stayed bridge in Hong Kong, where a Bayesian regression model was developed to characterize the relationship between temperature variation and expansion-joint displacement using long-term SHM data. The posterior predictive distribution was subsequently used to define a probabilistic anomaly index, and an alarm was generated when the probability of exceeding the predefined anomaly threshold reached a specified confidence level. This study represents an early demonstration of converting probabilistic monitoring outputs into actionable bridge management decisions. The anomaly index reflects expansion-joint behavioral deviation from a learned baseline. Alarm triggering is most appropriate for continuous online monitoring; its principal limitation is threshold calibration under posterior uncertainty and the false-alarm and missed-detection trade-off.

Any alarm system of this kind, however, is fundamentally dependent on the quality and uncertainty of the monitoring data used for posterior updating. Sensor noise, insufficient resolution, missing observations, and inappropriate sensor placement can introduce uncertainty into the estimated reliability index and may result in either false alarms or delayed detection of structural deterioration. Zorzi et al. [169] addressed this issue by introducing two reliability-based metrics, the Monitoring System Resolution (MSR) and the Monitoring System Importance Measure (MSIM), to quantify the influence of SHM data quality on reliability assessment. The MSR evaluates the sensitivity of the estimated reliability index to changes in monitored quantities; indicating how effectively a sensor system can resolve variations in structural reliability. In contrast, the MSIM identifies the relative contribution of each sensor measurement to the reliability update, providing guidance for sensor prioritization and monitoring system optimization. These concepts establish an important connection between sensor system design and the reliability of subsequent maintenance decisions, emphasizing that uncertainty in monitoring infrastructure directly propagates into uncertainty in structural management outcomes. The MSR and MSIM metrics quantify the information content of the monitoring system but do not constitute a decision rule. A complete alarm decision framework requires specification of the Type I and Type II error trade-off, the operating point on the ROC curve calibrated to the expected cost of false alarms against missed detections, and a lower credible bound on *β* and an upper credible bound on $P_{f}$ at a consistent probability level, rather than point estimates [99,100].

While single-bridge alarm systems demonstrate the feasibility of reliability-based decision-making, practical bridge management requires prioritization across networks where multiple structures may simultaneously exhibit reduced reliability under limited maintenance resources. Table 9 defines five alarm levels from sensor/data-quality through network prioritization; a Level 2 novelty alarm warrants investigation but does not constitute evidence of limit-state exceedance at Level 4. Orcesi and Frangopol [170] extended sensor-informed reliability assessment from individual structures to maintenance optimization by developing a framework that incorporated SHM information into lifecycle management decisions. Through Bayesian updating of time-to-failure probability density distributions, they demonstrated that sensor-informed maintenance scheduling reduces lifecycle costs compared with conventional strategies without monitoring information, using I-39 Northbound Bridge in Wisconsin as a case study. This work established the conceptual foundation for integrating reliability updating with network-level maintenance optimization.

Subsequent studies expanded this concept toward large-scale bridge management. Poli et al. [171] developed a prioritization framework for the Italian bridge network by ranking structures according to their proximity to predefined reliability thresholds ($\beta_{target}$), demonstrating how sensor-informed reliability indicators can support allocation of limited maintenance resources across large infrastructure portfolios. Brighenti et al. [172] further advanced this approach moving from reactive intervention strategies toward predictive maintenance through coupling of rapid reliability assessment with Markov-chain damage forecasting. Their framework estimated the future evolution of bridge reliability and identified the optimal timing for preventive interventions before the reliability index reached critical levels. Together, these studies illustrate the progression of sensor-informed maintenance strategies from single-bridge reliability monitoring to portfolio-level prioritization, and ultimately toward predictive lifecycle management. At network scale, satellite radar interferometry provides a complementary sensing layer enabling portfolio-level reliability prioritization without requiring instrumentation of individual bridges. The ReLUIS/DPC guidelines [57] establish criteria for integrating InSAR data with in-situ monitoring, providing a preliminary network-scale information layer that can inform prior distributions or prioritization for subsequent bridge-specific Bayesian updating. The ReLUIS bridge-specific volume [58] proposes systematic use of satellite radar data as a preliminary diffuse informative layer, complementary to conventional inspection and in-situ monitoring.

The applications discussed above primarily address the present reliability state by determining whether the current value of *β* has already reached or approached an intervention threshold $\beta_{target}$. However, effective infrastructure management requires a more proactive capability: predicting when reliability will deteriorate to an unacceptable level and evaluating how alternative maintenance strategies can delay this transition. This requirement leads naturally to RSL estimation problem, where temporal evolution of reliability is modeled using deterioration process informed by monitoring data.

### 4.2. Sensor-Informed Remaining Service Life Estimation

RSL represents the time interval between the present assessment and the point at which the reliability index, *β(t)*, is projected to decline to a predefined minimum acceptable level, $\beta_{target}$, beyond which the probability of failure becomes unacceptable, or maintenance intervention is required [173,174,175]. Unlike conventional design-stage RSL estimates, sensor-informed RSL prediction continuously incorporates SHM observations through Bayesian updating, allowing deterioration forecasts to evolve as new evidence becomes available. Consequently, RSL becomes a dynamic quantity that reflects the current structural condition rather than relying solely on deterministic deterioration assumptions. RSL estimation is most appropriate for long-term planning under stationary conditions; its principal limitation is sensitivity to the assumed deterioration model, as discussed in Section 4.2.1.

The foundation for probabilistic RSL estimation was established by Frangopol et al. [176], who represented the temporal evolution of the reliability index using a simple linear deterioration model:

(7) $$\beta \left(t\right)=\;\beta_{0}-\left(t-\;t_{i}\right)\cdot \alpha ,\;\mathrm{for}\;t>t_{i},$$

where *β*_0_ is the initial reliability index, $t_{i}$ is the time at which deterioration initiates, and α is the deterioration rate describing the reduction in reliability (*β*) per unit time. The RSL corresponds to the interval from the current assessment $t_{current}$ to the future crossing time t, where $\beta \left(t^{*}\right)$ = $\beta_{target}$; from Equation (7), t* = $t_{i}$ + (*β*_0_ *−* $\beta_{target}$)/α, giving RSL = ${t^{*}-t}_{current}$. In practice, *β*_0_, $t_{i}$, and α are themselves uncertain and are therefore modelled as random variables. Consequently, RSL is not represented by a single deterministic value but by a probability distribution reflecting uncertainty in the current structural condition, deterioration process, and future performance [176].

**Figure 4:**
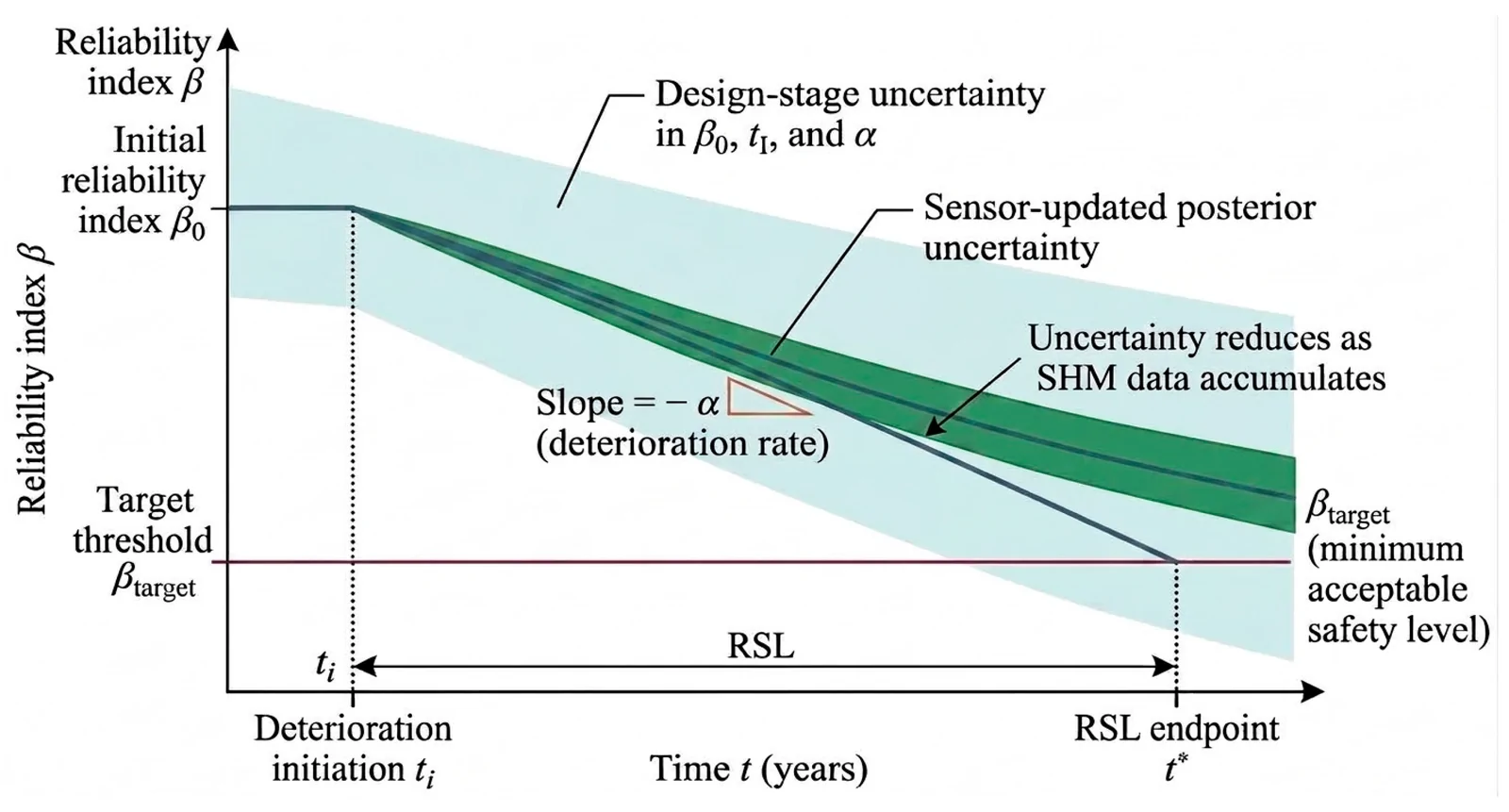
Sensor-informed RSL estimation based on Equation (7), showing the linear *β*(*t*) deterioration trajectory with uncertainty bands that narrow progressively as sequential Bayesian updating refining static parameter estimates under stationary assumptions; non-stationarity or model discrepancy can maintain or increase predictive uncertainty [173,176].

Figure 4 illustrates this concept schematically. Prior to monitoring, predictions of *β*(*t*) exhibit relatively wide uncertainty because they depend primarily on design assumptions about *β*_0_, $t_{i}$, and *α* (light shading).

As monitoring data accumulates, sequential Bayesian updating progressively refines the posterior distributions of the static parameters $t_{i}$, *β*_0_ and α, which can reduce epistemic uncertainty in the predicted trajectory under the stationary assumptions of Equations (7) and (8) [173,176]; in non-stationary systems, process uncertainty, forecast horizon, parameter evolution, and model discrepancy can maintain or increase predictive uncertainty. This progressive reduction in epistemic uncertainty enables maintenance interventions to be scheduled more effectively, avoiding both unnecessarily conservative decisions and delayed interventions that increase structural risk [176].

#### 4.2.1. Strengths and Limitations of the Linear Deterioration Model

The deterioration model proposed by Frangopol et al. [176] remains one of the most widely adopted frameworks for sensor-informed bridge reliability because of its analytical simplicity, computational efficiency, and straightforward physical interpretation. Its principal advantage is that the RSL prediction problem is reduced to three parameters, *β*_0_, $t_{i}$, and *α*, all of which can be recursively updated using Bayesian inference methods discussed in Section 3.1. This compatibility with Bayesian updating has made the model particularly attractive for long-term SHM applications, where reliability predictions must be revised repeatedly as new monitoring data become available.

The principal limitation of Equation (7), however, lies in its assumption of a constant deterioration rate (α). Real deterioration processes rarely evolve linearly. Chloride-induced corrosion typically accelerates after concrete cracking initiates. Fatigue crack growth follows nonlinear fracture mechanics, creep, and shrinkage evolve according to nonlinear time-dependent constitutive relationships, and prestress losses decrease nonlinearly throughout the service life of prestressed concrete bridges [52]. Consequently, although the linear deterioration model generally provided reasonable RSL predictions during the early stages of structural degradation, its accuracy progressively decreases as nonlinear damage mechanisms become dominant.

A further limitation is that Equation (7) cannot explicitly represent maintenance interventions. Practical bridge management rarely consists of uninterrupted deterioration; instead, inspection, repair, strengthening, or rehabilitation periodically modify the structural reliability trajectory. Minor maintenance may temporarily reduce the deterioration rate without restoring the original reliability level, whereas major rehabilitation can substantially increase *β* and effectively restart the deterioration process. To accommodate these effects, a piecewise linear extension incorporating maintenance effects is given by [176,177]:

(8) $$\beta \left(t\right)=\;\beta_{0}-\left(t-\;t_{i}\right)\cdot \alpha +\;\sum_{j}∆\beta_{j}\cdot \;H\left(t-\;t_{j}\right),\;\;$$

where $∆\beta_{j}$ is the reliability improvement from the *j*th maintenance intervention at time $t_{j}$, and *H*(·) is the unit step function. Unlike Equation (7), this formulation allows discontinuous increases in reliability following maintenance while preserving linear deterioration between intervention events.

Although Equation (8) provides a more realistic representation of maintenance planning, it still assumes linear deterioration during each interval between interventions. Complex deterioration driven by corrosion acceleration after cracking, nonlinear fatigue damage accumulation, creep–shrinkage interaction, and combined mechanical-environmental effects cannot be adequately represented within this framework and instead require physics-based deterioration models [18,52]. Stochastic process models provide the natural probabilistic alternative to the linear *β*(*t*) model of Equations (7) and (8). There are three most common models relevant to bridge deterioration from corrosion and fatigue. The gamma process models monotone non-decreasing deterioration through independent non-negative gamma increments; van Noortwijk [178] showed it is the natural model for corrosion depth, section loss, and fatigue crack growth, providing the standard reference for its application to inspection and maintenance. Frangopol et al. [179] connected this framework directly to bridge life-cycle performance. RSL is obtained from the first passage time distribution, that is the time at which cumulative deterioration exceeds the failure threshold, providing a physically consistent alternative to Equation (7). The Wiener process models bidirectional degradation; Whitmore [180] established it for degradation subject to measurement error, with first passage time following the inverse Gaussian distribution from which RSL is computed analytically. The inverse Gaussian process, introduced by Ye and Chen [181], provides a flexible middle ground with non-negative right-skewed increments suited to high-variability deterioration. All three processes can be updated sequentially via Bayesian filtering as monitoring data accumulate, replacing the fixed *α* and *β*_0_ of Equation (7) with narrowing posterior distributions [178,179].

Beyond individual deterioration mechanisms, bridge reliability assessment increasingly recognizes that deterioration is fundamentally a system-level problem. Foundation scour, for example, requires time-dependent failure probability models for piers [160], while bearing degradation evolves nonlinearly under repeated traffic loading and environmental exposure [182]. Likewise, the RSL is governed by the interaction among multiple structural elements. Consequently, component-level Bayesian updating must ultimately be integrated within system reliability frameworks to estimate bridge-level RSL and support network-scale maintenance planning [118,121]. System reliability analysis provides a formal framework for this integration. At the component level, individual member failure probabilities are computed from the posterior distributions of resistance and load-effect parameters. At the system level, the bridge is modelled as a combination of series and parallel subsystems: a series system fails when any one component fails, while a parallel system fails only when all components fail simultaneously [183]. For systems with many failure modes, exact computation of the system failure probability is intractable. Ditlevsen bounds provide narrow upper and lower bounds on the system failure probability using only component failure probabilities and pairwise joint probabilities, without enumerating all failure modes [183]. The matrix-based system reliability method of Kang et al. [184] represents system events in matrix form and estimates the narrowest bounds on system failure probability by linear programming, even under incomplete information on component probabilities and statistical dependence. Kang et al. [184] demonstrated the method specifically on bridge networks, making it directly applicable to the network-scale maintenance planning claimed in the conclusions. Sensor-informed Bayesian updating at the component level propagates through the series-parallel system model to update the system-level failure probability, connecting individual sensor observations to bridge-level RSL and network-scale reliability. At the network level, resilience quantifies the ability of a transportation network to maintain functionality and recover from disruptions. Frangopol and Bocchini [185] proposed resilience as the optimization criterion for the rehabilitation of bridges within a transportation network, directly linking component-level SHM data to network-scale management decisions.

#### 4.2.2. Sensor-Updated RSL with Bayesian Uncertainty Propagation

The principal contribution of SHM to RSL estimation is not the deterioration model itself, but the ability to continuously reduce uncertainty in its governing parameters through Bayesian updating. Rather than relying on prior distributions derived from design assumptions or historical deterioration models, sequential sensor observations progressively update the posterior distributions of the *β*_0_, $t_{i}$, and *α*. Consequently, the uncertainty associated with the predicted reliability trajectory decreases over time, producing increasingly reliable estimates of remaining service life as monitoring data accumulate.

Alam et al. [173] demonstrated this capability using a Bayesian probability-box (p-box) framework that explicitly distinguishes between aleatory uncertainty arising from the inherent randomness of deterioration processes and epistemic uncertainty resulting from incomplete knowledge of the structural condition. Applied to a reinforced concrete bridge subjected to corrosion deterioration, the framework showed that epistemic uncertainty associated with limited inspection could alter the estimated probability of failure by a factor of nearly two. More importantly, sequential Bayesian updating from recurrent inspection and monitoring data efficiently reduced the epistemic uncertainty bounds, producing progressively narrower confidence bounds on RSL prediction. Rather than providing a single deterministic service-life estimate, the method supplied bridge managers with a probabilistic range whose uncertainty decreased as additional structural evidence became available.

The dynamic nature of sensor-informed RSL prediction was further demonstrated by Qu and Sun [167], who combined long-term SHM stress measurements with a Bayesian dynamic regression model to continuously update the reliability deterioration trajectory. Unlike the linear deterioration framework of Equation (7), where the deterioration rate (*α*) is treated as a fixed parameter, the Bayesian formulation continuously revised α as new monitoring data became available. Consequently, predicted RSL evolved with the observed structural behavior, allowing deterioration trends that deviated from design assumptions to be identified early. This capability represents a significant advancement over conventional deterministic approaches, enabling maintenance schedules to respond to the actual deterioration history rather than assumed deterioration rates.

#### 4.2.3. Deflection and Prestress Sensor Applications to RSL

For prestressed concrete bridges, sensor-informed updating can fundamentally alter estimates of RSL because the dominant uncertainty frequently resides in the resistance model rather than the applied loading. Sarmiento et al. [52] illustrate this on the Kalix Bridge by integrating residual prestress measurements and material property testing within a Bayesian updating framework. The updated distribution increased the reliability index from a prior of 0.78 to a posterior of 2.84 at year 66 (50-year reference period, serviceability limit state [52]), a difference of over two reliability index units. The *β* values quoted across different studies are not directly comparable unless the reference period, limit state, and consequence class coincide. From an RSL perspective, this result is particularly significant because a bridge that appears to have reached its service life based on design assumptions may retain substantial remaining capacity once monitoring evidence is incorporated. Conversely, monitoring may also reveal deterioration progressing more rapidly than anticipated, indicating that intervention is required sooner than predicted by conventional deterioration models. This study highlights that sensor-informed RSL estimation is not merely a refinement of prediction accuracy but can fundamentally change maintenance decisions and asset management priorities.

#### 4.2.4. Component-Level Service Life Estimation

RSL estimation ultimately depends on monitoring the deterioration mechanisms governing individual structural components. Consequently, the selection of sensor technology should be matched to the dominant deterioration process rather than applying a uniform monitoring strategy across the entire bridge. Wei, Yi et al. [182] demonstrated this principle for bridge bearings by combining high-frequency accelerometer measurements, which captured dynamic displacement under vehicle loading, with low-frequency displacement sensor data to capture static cumulative displacement under temperature effects. Using cumulative displacement as the governing deterioration indicator, the probability that cumulative displacement exceeds the allowable wear limit was translated directly into a component-level reliability measure and corresponding service life prediction. This study illustrates a broader principle for sensor-informed bridge management: deterioration mechanisms, monitoring technologies, and reliability metrics should be selected consistently for each structural component. Bearings, cables, decks, girders, foundations, and expansion joints experience different degradation processes and therefore require different sensing strategies and limit-state formulations. Consequently, component-level RSL estimation should be interpreted within the context of the governing deterioration mechanism rather than being directly extrapolated to the entire bridge system.

#### 4.2.5. Practical Engineering Applicability of Sensor-Informed RSL

The practical value of sensor-informed RSL estimation depends largely on the availability of long-term, high-quality monitoring data. Continuous monitoring enables progressive refinement of deterioration parameters through Bayesian updating, whereas short or discontinuous monitoring records leave substantial uncertainty in the posterior distribution of the deterioration rate *α*, resulting in wide confidence intervals for predicted RSL. Consequently, sensor observations should be complemented by periodic inspections and condition assessments to improve parameter identification and reduce predictive uncertainty [173].

Current SHM systems typically monitor specific critical components rather than the entire structure, as demonstrated for cable-stayed and truss bridges [118]. Accordingly, most existing sensor-informed RSL estimates should be interpreted as component-level predictions rather than whole-bridge service life assessments. In engineering practice, the linear deterioration model represented by Equation (7) serves as an effective first-order screening tool for identifying structures approaching critical reliability thresholds, while nonlinear deterioration models provide greater predictive accuracy for aging bridges exhibiting complex degradation mechanisms [52,176].

It is also important to distinguish between short-term operational alarms and long-term lifecycle predictions. Reliability thresholds used for immediate condition monitoring are intended to detect abnormal structural behavior requiring operational attention, whereas lifecycle maintenance decisions require comprehensive Bayesian reliability analysis that incorporates deterioration modelling, consequence assessment, maintenance effectiveness, and economic optimization. Although both decisions rely on the reliability index *β*, they address fundamentally different engineering objectives and therefore should not be interpreted interchangeably [173,176].

#### 4.2.6. Open Challenges in Sensor-Informed RSL Estimation

Despite substantial progress, several important challenges remain before sensor-informed RSL estimation can be routinely implemented for bridge asset management.

The first challenge concerns the propagation of monitoring uncertainty throughout the Bayesian prediction framework. Although preprocessing techniques reviewed in Section 2 address the sensor imperfections, few studies explicitly quantify how these uncertainties propagate through Bayesian updating into the posterior distributions of deterioration parameters and, ultimately, into predicted RSL. End-to-end uncertainty propagation therefore remains insufficiently addressed in current bridge RSL applications.

A second challenge is the representation of multiple interacting deterioration mechanisms. Most existing RSL models assume a single dominant deterioration process, whereas real bridges simultaneously experience corrosion, fatigue, creep, shrinkage, scour, bearing deterioration, and environmental degradation. Developing unified Bayesian frameworks capable of integrating heterogeneous sensor observations and jointly modelling these coupled deterioration mechanisms remains an important research direction.

Finally, uncertainty remains regarding the definition of the target reliability threshold, $\beta_{target}$, used for lifecycle prediction. Existing threshold values are generally derived from design-stage reliability requirements, yet monitoring-informed reliability assessments may differ substantially from those assumptions because they incorporate the actual structural condition. Consequently, there is currently no universal consensus regarding the most appropriate reliability threshold for sensor-informed maintenance planning across different bridge types, deterioration mechanisms, and regulatory frameworks [173,176]. Several normative frameworks provide $\beta_{target}$, values that sensor-informed assessments should reference. ISO 2394 [2] and ISO 13822 [3] define target reliability indices for structural design and assessment of existing structures respectively; EN 1990 [186] covers the European design framework; fib Bulletin 80 [4] provides partial factor methods for existing concrete structures that explicitly incorporate monitoring information; the JCSS Probabilistic Model Code [187] provides the underlying probabilistic basis; and the AASHTO Manual for Bridge Evaluation [188] explicitly distinguishes design-stage reliability targets from in-service evaluation targets. Target reliability values depend on the reference period, the consequence class, the cost of safety measures, and whether assessment is at component or system level. *β* values reported across studies are therefore not directly comparable unless the reference period and limit state coincide, and this dependence must be acknowledged when interpreting sensor-informed reliability updates across different regulatory contexts. Establishing monitoring-informed reliability criteria that are scientifically rigorous and compatible with these normative frameworks remains a key research challenge.

### 4.3. Reliability-Based Lifecycle Maintenance Optimization

Sensor-informed reliability assessment reaches its practical objective when updated reliability predictions are incorporated into lifecycle maintenance planning. Rather than using reliability indices solely as indicators of structural safety, lifecycle optimization uses sensor-updated reliability trajectories and RSL projections to determine the optimal timing, type, and sequencing of maintenance interventions while satisfying safety, cost, and resource constraints. In this framework, Bayesian updating provides a probabilistic description of the current structural condition, whereas optimization identifies maintenance strategies that minimize expected lifecycle costs without compromising the target reliability level. Lifecycle optimization is most appropriate when full cost–benefit analysis is required; its principal limitations are computational cost and the need for well-specified cost models.

The conceptual foundation for this framework was established by Frangopol et al. [176], who demonstrated that maintenance interventions modify the temporal evolution of the reliability index by either restoring structural reliability toward its initial level or reducing the subsequent deterioration rate α. The economic lifecycle optimization problem can be formulated as:

(9) $$\mathrm{minimize}\;C_{total}=\;C_{initial}\;+\;C_{maintenance}\;+\;C_{failure},\;\mathrm{subject}\;\mathrm{to}:\;\beta (t)\;\geq \;\beta_{target},\;t\;\in \;[0,\;T]$$

where $C_{initial}$, $C_{maintenance}$, and $C_{failure}$ are present-value cost terms and *T* is the planning horizon. Equation (9) is a minimal conceptual expression adapted from the lifecycle cost framework of Frangopol et al. [176]. A complete formulation would additionally include discounting, expectations over the posterior predictive state, and a probabilistic rather than deterministic safety constraint. Within this framework, SHM contributes by continuously updating the reliability trajectory through Bayesian inference, replacing conservative design-stage assumptions with posterior distributions conditioned on observed structural behavior. As uncertainty in *β*(*t*) decreases, intervention timing can be optimized more accurately, reducing unnecessary maintenance while maintaining acceptable levels of structural safety.

#### 4.3.1. Advantages of Sensor-Informed Lifecycle Optimization

The primary advantage of sensor-informed lifecycle optimization lies in replacing generic deterioration assumptions with bridge-specific reliability predictions derived from monitoring evidence. Instead of scheduling maintenance according to bridge age or condition ratings, interventions can be prioritized using the actual probabilistic safety state of the structure. Consequently, maintenance decisions become adaptive, reflecting the observed deterioration history rather than expected deterioration derived from design assumptions.

As demonstrated by Poli et al. [171] and Brighenti et al. [172] in Section 4.1, this advantage derives from the quality of the probabilistic information driving the decision process rather than from the optimization algorithm itself. More informative monitoring data produces narrower posterior distributions of *β*(*t*), resulting in more accurate RSL predictions, better-timed interventions, and ultimately lower total lifecycle costs.

#### 4.3.2. Limitations and Practical Constraints of Sensor-Informed Lifecycle Optimization

Despite these advantages, several practical limitations continue to restrict the widespread implementation of sensor-informed lifecycle optimization. The first is computational complexity. Bayesian updating of large bridge models containing numerous uncertain structural parameters remains computationally intensive, particularly when high-fidelity finite element models are coupled with reliability analysis. Although surrogate models, variational inference, and sequential filtering methods substantially improve computational efficiency, these approximations may underestimate posterior uncertainty, particularly in the tails of the distribution where lifecycle optimization is most sensitive.

A second limitation concerns deterioration modelling. The quality of the optimized maintenance strategy depends directly on the accuracy of the deterioration model used to predict future reliability. If the assumed deterioration mechanism is inconsistent with the actual physical behavior, for example, adopting a linear deterioration model for a bridge dominated by nonlinear fatigue crack propagation or corrosion acceleration, the resulting maintenance schedule may be suboptimal regardless of the quality of the monitoring data.

Finally, lifecycle optimization depends on accurate characterization of economic parameters, including maintenance costs, inspection costs, failure consequences, discount rates, and intervention effectiveness. These quantities vary substantially among bridge owners, structural systems, and geographical regions, limiting the direct transferability of optimization results between infrastructure networks [176]. The formulation of Equation (9) treats lifecycle optimization as a static cost minimization problem, whereas inspection and maintenance decisions are made sequentially under uncertainty as monitoring data accumulate. This class of problems is formulated as a partially observable Markov decision process (POMDP), in which the optimal policy maps beliefs about the structural state to inspection and maintenance actions, explicitly capturing the economic value of future monitoring information through adaptive intervention policies. SHM changes the optimal policy by updating the belief state over structural condition, replacing the deterministic constraint $\beta \left(t\right)\geq \beta_{target}$ with a probabilistic constraint on the posterior predictive failure probability and shifting intervention timing from schedule-based to condition-based. Papakonstantinou and Shinozuka [189,190] established the POMDP framework for structural inspection and maintenance planning, showing that optimum policies incorporating the cost–benefit of information cannot be matched by simpler Markov decision process approaches. Memarzadeh and Pozzi [191] extended this to integrated inspection scheduling and maintenance planning, explicitly incorporating the value of monitoring information. The principal limitation of POMDP methods is poor scalability with system size; Andriotis and Papakonstantinou [192] addressed this through deep reinforcement learning, presenting the deep centralized multi-agent actor–critic approach for large-scale multi-component systems under partial observability. These developments represent the current research frontier of sensor-informed lifecycle optimization.

### 4.4. Value of Information (VoI) of SHM Systems

A distinct but closely related question is whether the monitoring system itself provides sufficient economic benefit to justify its installation and operation. This is addressed through the value-of-information (VoI) analysis, which quantifies the reduction in expected lifecycle costs attributable to monitoring-informed decision-making relative to conventional maintenance strategies.

Valkonen and Glisic [193] investigated this problem by combining neural network-based survival analysis with Monte Carlo simulation to evaluate the lifecycle extension benefits of SHM for a concrete bridge deck on US Highway 202 in Wayne, New Jersey. Their analysis estimated that the present value of postponed bridge replacement cost resulting from SHM-informed maintenance ranged from approximately USD 8.0 million to USD 39.0 million, depending primarily on replacement cost assumption. Sensitivity analysis further revealed that the discount rate exerts an equally large effect on the realizable VoI, emphasizing that economic assumptions can be as important as structural performance in determining the value of monitoring investments. These results demonstrate that, under appropriate conditions, the economic benefits of an SHM can substantially exceed deployment and operational costs.

The economic value of monitoring, however, is highly bridge-specific. VoI depends on numerous factors, including deterioration mechanisms, bridge importance, maintenance strategy, inspection costs, replacement costs, monitoring reliability, and the quality of the acquired sensor data. Kamariotis et al. [66] further demonstrated that VoI is strongly influenced by the informativeness of the sensor configuration itself. Using a numerical model of a two-span bridge, they showed that monitoring systems providing greater information about deterioration-related parameters produce larger lifecycle benefits. This finding complements the MSR and MSIM proposed by Zorzi et al. [169], linking sensor placement and monitoring system design directly to lifecycle economic performance. Straub [194] established the formal framework for VoI analysis using structural reliability methods, showing how pre-posterior decision analysis quantifies the expected benefit of monitoring before implementation. Thöns et al. [195] applied this to fatigue deteriorating structural systems. Zonta et al. [196] demonstrated measurable VoI impact on bridge maintenance decisions. Malings and Pozzi [197] extended VoI to spatially distributed systems, connecting it directly to optimal sensor placement. COST Action TU1402 (2014–2019), a 29-country network devoted specifically to quantifying the value of SHM, constitutes the most extensive collaborative treatment of this topic to date [198]. Together these works establish VoI as a formal pre-posterior decision analysis tool that should guide sensor placement, monitoring duration, and inspection scheduling from the outset.

### 4.5. Current Trends and Future Outlook

Four major trends characterize the evolution of sensor-informed bridge lifecycle management.

First, maintenance optimization is shifting from individual bridge applications toward portfolio-scale and network-level asset management, where reliability information from multiple structures is integrated to support inspection prioritization and maintenance budget allocation across entire transportation networks [171,172]. The integration of satellite radar interferometry into bridge management frameworks is expected to expand further as data archives lengthen and fusion algorithms with in-situ monitoring mature [56,57,58].

Second, predictive maintenance is gradually replacing conventional reactive maintenance strategies. Rather than responding only after reliability has deteriorated below acceptable levels, modern SHM systems increasingly forecast future *β*(*t*) trajectories and RSL, allowing maintenance interventions to be scheduled proactively before critical reliability thresholds are reached [172].

Third, VoI is emerging as an integral component of SHM planning. Rather than assuming that monitoring is always beneficial, recent studies explicitly quantify whether the expected reduction in maintenance costs and failure risk justifies the investment required for sensor deployment and long-term operation [66,193].

Fourth, the digital twin is emerging as the prominent framing for the model-plus-monitoring architecture that this review describes. A digital twin is a continuously updated virtual representation of a physical structure linked to the physical asset through bidirectional sensor data flows [199,200]. Ghahari et al. [165] demonstrated bridge digital twinning through sequential Bayesian model updating with recorded seismic measurements. As computational costs fall and real-time model updating becomes feasible, digital twins represent a promising but not yet operationally established platform; widespread deployment remains subject to computational cost, data governance, and model validation barriers.

Taken together, these developments show increasing integration of sensor-informed reliability assessment with predictive maintenance, value of information analysis, and digital-twin frameworks. However, sustained operational deployment in bridge asset management remains limited.

## 5. Discussion

The integration of sensor data into probabilistic bridge reliability analysis has advanced substantially, yet a persistent gap remains between monitoring technologies and their rigorous integration into reliability assessment. Sensor-informed reliability is not a direct mapping from raw measurements to safety indices but an interconnected chain in which sensor imperfections, preprocessing choices, model discrepancy, and inference assumptions propagate into reliability indices and RSL estimates.

A key gap concerns the sensor to reliability pathway. Monitoring observations do not directly determine *β* but require a defensible forward model linking measured quantities to limit state variables, together with consideration of parameter identifiability and spatial representativeness. Similarly, multi-sensor fusion can increase information content, but its benefits depend on sensor reliability, error correlation, model adequacy, and appropriate spatial and temporal alignment.

Preprocessing constitutes a reliability-critical rather than a neutral data cleaning step. Filtering, thermal compensation, anomaly detection, missing data imputation, and calibration influence the uncertainty carried into subsequent Bayesian inference; residual uncertainty should therefore be characterized and propagated rather than treating processed observations as error free. The illustrative scenario in Table 3 demonstrates this propagation, with RSL effects following the ordering drift > missing data > bias; however, this ordering is conditional on the assumed parameter values and monitoring conditions and does not represent a general ranking of sensor imperfections.

No single inference method is universally suitable, as method selection depends on likelihood availability, model complexity, data characteristics, and the intended reliability application. Hybrid Bayesian reliability frameworks provide a useful connection between posterior inference and engineering reliability metrics, although sustained operational implementation for code-compliant assessment remains limited. Alarm levels must also be clearly distinguished: sensor data quality flags, response anomalies, damage state inferences, component limit state reliability alerts, and network prioritization decisions each require different decision variables, thresholds, and verification procedures.

The significance of sensor-informed reliability extends beyond *β* estimation to RSL prediction, maintenance planning, value of information analysis, and lifecycle decision making. Six barriers currently constrain routine deployment: data governance, model validation, computational certification of inference methods, sensor maintainability, decision accountability, and regulatory acceptance. Addressing these would strengthen continuous monitoring as defensible evidence for bridge reliability and lifecycle management.

## 6. Conclusions

This review examined the complete workflow of sensor-informed probabilistic reliability assessment for aging bridge structures, from sensor data acquisition and preprocessing to Bayesian uncertainty updating, reliability evaluation, RSL prediction, and lifecycle maintenance optimization. The following conclusions are drawn:

1. Sensor selection consistently emerges as a primary determinant of the reliability information that can be extracted from monitoring data. No single sensor technology is universally optimal; rather, sensor selection must be aligned with the target reliability variable and governing deterioration mechanism. Strain-based sensors provide direct information for load-effect characterization, and corrosion and prestress-related measurements inform resistance deterioration, while vibration and vision-based systems support damage identification and state updating. Effective reliability assessment therefore requires balancing measurement directness, long-term stability, and preprocessing requirements.
2. Consistent evidence across preprocessing, inference, and reliability studies demonstrates that data quality is a fundamental determinant of reliability prediction accuracy. Sensor bias, drift, thermal effects, noise, and missing observations propagate through preprocessing, Bayesian updating, reliability index estimation, and RSL prediction. The relative severity of these imperfections depends on their specific magnitudes: the worked example in Table 3 illustrates one scenario in which drift dominates, but missing data or bias may govern under different monitoring conditions. Therefore, preprocessing should be considered an integral component of the reliability framework rather than a separate data-cleaning step. Future developments should prioritize end-to-end uncertainty quantification from sensor to reliability index, physics-informed deterioration models for non-stationary degradation, scalable inference validated for network-level deployment, and standardized protocols enabling code-compliant reliability certification from operational monitoring data.
3. Bayesian inference provides the essential framework for converting monitoring data into probabilistic reliability metrics. Among the reviewed approaches, five core posterior inference methods and four supporting inference methods are compared in Table 5 and Table 6 respectively. Hybrid Bayesian–reliability frameworks provide the closest conceptual connection between field observations and actionable safety indicators, though most reviewed studies demonstrate this at the numerical or laboratory validation stage rather than through sustained operational deployment. While the framework is broadly agreed upon, consensus on which specific method is most appropriate for code-compliant assessment has not yet been reached. Computational cost remains a key barrier for large-scale deployment, motivating increased use of surrogate models, particle-based approaches, and advanced reduced-order methods.
4. Field studies consistently demonstrate sensor-informed reliability can substantially modify lifecycle decisions compared with design-stage assumptions. Monitoring evidence can revise reliability estimates, extend or reduce predicted service life, and support optimized maintenance strategies. Value-of-information studies suggest that SHM investments can be economically justified when monitoring reduces uncertainty and enables better-timed interventions. Nevertheless, practical deployment requires addressing three remaining challenges: (i) systematic propagation of sensor and preprocessing uncertainty into RSL predictions, (ii) multi-mechanism deterioration modeling under heterogeneous sensor networks, and (iii) consistent reliability thresholds for monitoring-informed assessments, (iv) model validation and computational certification of inference methods for regulatory use, and (v) decision accountability and regulatory acceptance frameworks.

In summary, sensor-informed probabilistic reliability assessment spans a wide maturity spectrum: conceptual methods and numerical demonstrations are well established, while online pilots and sustained operational decision use remain rare. Sensor preprocessing, Bayesian model updating, and reliability index estimation in retrospective field studies have been validated; formal code-compliant *β* estimation in operational settings has not yet been demonstrated. Sustained online updating, network-scale deployment, and code-compliant reliability certification remain prospective. The future of bridge reliability lies in combining high-quality multi-source sensing, advanced Bayesian uncertainty quantification, physics-informed deterioration models, and lifecycle decision analytics to enable evidence-based and economically efficient management of aging bridge networks.

## Figures and Tables

**Table 1:** Scope comparison of the present review with key prior reviews.

Study	Sensor Technologies	Data Quality & Preprocessing	Bayesian Inference	Reliability Index (β)	RSL & Maintenance	Integrated Reliability Pipeline
Figueiredo & Brownjohn [27]	✓	✓	–	–	–	–
Deng et al. [21]	✓	✓	–	–	–	–
Bao et al. [28]	✓	✓	–	–	–	–
Rizzo & Enshaeian [7]	✓	–	–	–	–	–
Tang et al. [29]	✓	–	✓	–	–	–
Teh et al. [26]	✓	✓	–	–	–	–
Primo et al. [18]	–	–	–	✓	✓	–
Malekjafarian et al. [30]	✓	–	–	–	–	–
Simoen et al. [31]	–	✓	✓	–	–	–
Macchiarulo et al. [32]	✓	–	–	–	–	–
**This study**	✓	✓	✓	✓	✓	✓

Note: Yes (✓) = primary focus with a dedicated section; No (–) = not addressed or incidental. Integrated Pipeline = integrated coverage from sensor data quality through Bayesian inference, reliability index *β*, RSL estimation, and maintenance optimization. Comparator reviews were selected from the same structured narrative search to represent prior works addressing at least one pipeline stage. Several reviews partially overlap with individual stages; the table situates the scope of this review rather than establishing exhaustiveness.

**Table 2:** Summary of sensor technologies and their roles in bridge reliability assessment.

Sensor Technology	Measured Quantity/Parameter	Latent Reliability Variable/Transfer Model	Key Strength/Temporal–Spatial Resolution	Key Limitations/Principal Error Sources	Validation Needs
Strain Gauges (resistive/vibrating wire) [34,35,36]	Local strain/stress	Load-effect PDF *S*(*t*); fatigue Δσ/FORM; load-effect model	Load-effect measurement; directness depends on limit state and installation; Hz–kHz; point measurement	Drift; thermal effects; debonding; calibration errors	Reference load calibration; cross-sensor consistency
Corrosion Sensors [37,38,39]	Electrochemical parameters; corrosion current; FBG-based strain indicator	Section loss *R*(*t*); initiation time; deterioration rate *α*/Deterioration model	Direct deterioration measurement; low temporal; point	Spatially varying corrosion missed; durability concerns; humidity effects	Physical inspection cross-check; destructive testing
Fiber Bragg Grating (FBG) [40,41]	Strain, temperature, displacement	Temperature-compensated strain; load-effect distribution/Temperature compensation model	Quasi-distributed; EM immunity; long-term stable; moderate temporal	High cost; fragile; temperature-strain cross-sensitivity	Electrical strain gauge comparison; thermal calibration
Piezoelectric (PZT) Sensors [42,43,44]	Impedance; acoustic emission; strain	Stiffness degradation; damage features/Impedance-damage correlation model	Sensitive to incipient damage; embeddable; high frequency; point	Baseline dependent; temperature sensitive; limited sensing range	Laboratory damage simulation; visual inspection comparison
Accelerometers [45,46]	Vibration, modal frequencies	Modal frequencies; stiffness *K*(*t*); model parameters θ/FEM updating; OMA	Continuous SHM; low cost; mature; directness depends on limit state and installation; high temporal; sparse spatial	Indirect inference; noise in low-cost sensors; temperature effects,	Multi-chain MCMC diagnostics; known damage cross-validation
GNSS [47,48]	Absolute displacement	Long-term settlement; global deformation; serviceability/Settlement model	Drift-free global; no physical reference; low temporal (1 Hz); global	Low sampling rate; multipath errors in complex environments	Total station survey; levelling benchmarks
Temperature & Humidity Sensors [7]	Temperature; humidity	Environmental covariate for normalization only/Regression normalization	Essential for separating environmental from structural; moderate temporal; point	No direct reliability update without structural sensors	Independent weather station comparison
Vision-based/DIC [49]	Full-field displacement, crack width	Crack width; deformation field; damage state/Image processing; crack growth model	Non-contact; full-field; high spatial; moderate temporal	Line-of-sight required; illumination sensitivity; occlusion	Contact sensor comparison; laboratory crack measurement
WIM [50]	Axle load; vehicle weight; speed	Site-specific live-load PDF *S*(*t*)/Traffic load model; extreme value extrapolation	Replaces code-based traffic; continuous temporal; single lane	Site-specific; calibration drift; limited transferability	Weighbridge calibration; design traffic model comparison
Deflection Transducers [51,52]	Structural deflection	Integrated deformation from creep, shrinkage, prestress, support effects/Calibrated structural model to separate contributions	Global structural performance; moderate temporal; point or limited spatial	Stable reference required; combined effects inseparable without structural model	FEM prediction comparison; staged loading tests
Anemometer [53,54]	Wind speed, direction, turbulence	Wind-induced load effect *S*-wind (*t*); flutter parameters/Aerodynamic model	Essential for wind-sensitive bridges; high temporal; point	Wind-sensitive bridges only; aerodynamic model required	Wind tunnel comparison; aerodynamic coefficient verification
Distributed Fibre Optic Sensors (DFOS) [55]	Continuous strain and temperature along fibre	Distributed *S*(*t*) profile; spatial deterioration/Distributed load model; temperature-strain separation	Truly distributed; no discrete gaps; lower temporal than FBG	Higher cost than FBG; limited spatial resolution; temperature-strain separation required	Discrete gauge comparison; load test validation
Satellite InSAR (DInSAR/SBAS) [56,57,58]	Displacement (line of sight)	Network-scale displacement history; preliminary information layer for bridge-specific updating/Two-stage hierarchical fusion; atmospheric correction	Wide-area; no installation; retrospective coverage; low temporal (revisit)	Line-of-sight only; atmospheric noise; thermal confounding; scatterer attribution	GNSS cross-validation; in-situ sensor comparison
Drive-by/indirect monitoring [30]	Vehicle dynamic response	Bridge frequency; condition indicator; modal parameters/Vehicle–bridge interaction model	No installation; network-scale screening; episodic	Indirect inference; vehicle–bridge uncertainty; speed sensitivity	Direct accelerometer comparison; controlled vehicle tests
Tiltmeters/Inclinometers [59]	Rotation; inclination	Pier rotation; settlement angle; scour-induced tilt/Structural deformation model	Direct measurement; prescribed in practice; moderate temporal; point	Rotation only; dynamic movement effects; temperature sensitivity	Survey measurement comparison; controlled loading tests
Crack meters [21,33]	Crack width and rate	Resistance degradation *R*(*t*); cracking progression/Fracture mechanics model	Direct crack measurement; moderate temporal; point only	Point measurement only; crack orientation sensitivity	Visual inspection comparison; laboratory fracture tests
Scour devices [60]	Scour depth; streambed level	Foundation capacity (*R*) scour; scour risk/Foundation capacity model	Direct scour measurement; continuous temporal; local	Debris vulnerability; limited spatial coverage; sensor burial	Bathymetric survey cross-validation; post-flood inspection
Magneto-elastic/load cells [61]	Cable force; tendon prestress	Prestress loss; cable force reliability/Force-reliability model	Direct force; high accuracy; high temporal; discrete points	Difficult installation in existing structures; temperature effects	Hydraulic jack comparison; destructive testing
Ground-based Radar [62]	Dynamic displacement; vibration	Modal parameters; deformation; serviceability/Structural identification model	Remote; no contact; sub-mm sensitivity; high temporal; line-of-sight	Line-of-sight only; atmospheric effects; noise floor	Contact displacement sensor comparison; accelerometer cross-validation
UAV photogrammetry [63]	Surface displacement; crack maps	Visual damage state; geometric deformation/Photogrammetric reconstruction; damage-reliability model	Wide area; high resolution; difficult access; episodic	Weather dependent; post-processing required; limited temporal	Close-range inspection comparison; contact crack measurement
Wireless/MEMS networks [45,46]	Acceleration; strain; temperature	Modal parameters; load effects/Structural identification model	Low cost; scalable; easy installation; moderate temporal	Lower accuracy than wired; power constraints; data loss	Wired reference sensor comparison; laboratory calibration

**Table 3:** Effect of sensor drift, bias, and MNAR missing data on the apparent reliability index *β* and remaining service life RSL. *β* values are conditional plug-in estimates from Equation (5) rather than posterior predictive quantities. Resistance, variance, temporal dependence, deterioration rate, and model uncertainty are held fixed. Positive RSL change = unconservative; negative RSL change = conservative.

Scenario	Load Effect *S*		Reliability Index (β)	RSL (yr)	RSL Change
	${µ}_{S}(MN)$	$\sigma_{S}(MN)$			
Baseline	2.500	0.500	2.77	8.0	0%
Drift (−ve)	2.250	0.500	3.05	16.2	+101%
Drift (+ve)	2.750	0.500	2.50	−0.1	−101%
Bias (−ve)	2.375	0.500	2.91	12.1	+52%
Bias (+ve)	2.625	0.500	2.63	4.0	−50%
Missing data	2.403	0.422	3.02	15.2	+91%
Combined (−ve)	2.028	0.422	3.45	28.1	+251%
Combined (+ve)	2.778	0.422	2.58	2.4	−70%

**Table 4:** Comparison of sensor data preprocessing methods and their implications for bridge reliability.

Preprocessing Method	Reliability Contribution	Reliability Limitation	Residual Uncertainty Source	Bridge Applications
Statistical filtering and denoising	Reduces measurement noise while preserving structural characteristics; MODWT provides superior performance under high-intensity white noise compared with time- and frequency-domain approaches	Excessive filtering may remove damage-related features; wavelet basis and parameter selection influence extracted response characteristics.	Method-dependent residual noise and potential signal distortion affecting parameter identification	MODWT applied to Marsh Lane and KW51 Bridges [82]; wavelet denoising applied to HZMB tunnel strain measurements [21,28].
Anomaly detection and outlier handling	Distinguishes genuine structural anomalies from data artefacts; enables automated real-time structural warning. Improves reliability of automated monitoring by preventing false alarms and erroneous Bayesian updates	No detection method achieves 100% accuracy; deep learning detection on a cable-stayed bridge achieved 85.6% accuracy, leaving undetected [81]; sensitivity, specificity, and precision are more appropriate metrics.	Remaining false negatives/false positives that may bias damage probability and reliability estimation	Deep learning-based anomaly detection for cable-stayed bridge [81]; ARIMA hierarchical warning on HZMB strain records [21]
Bias correction, thermal compensation, and calibration updating	Enables temperature-compensated structural responses for load-effect distribution fitting and long-term reliability assessment	Uncorrected environmental effects may exceed 10% of monitored responses and produce false damage [27].	Residual uncertainty in separate structural responses, especially under non-uniform temperature gradients.	PCA and regression-based thermal compensation [27,28]; Bayesian calibration updating [77].
Missing data imputation	Bayesian dynamic regression and learning-based methods reconstruct missing data while quantifying uncertainty; on cable-stayed bridges; reliable performance demonstrated for high random missing rates and moderate structured gaps	Performance degrades for long continuous gaps and highly structured missing patterns [98]	Reconstruction uncertainty depends on missing duration, missing pattern, and model	BDR for cable-stayed bridges [27]; WGAIN-GP for Tongling bridge [86]; tensor learning for concrete bridges [98].
Calibration updating	Online calibration updating maintains sensor accuracy over multi-year monitoring campaigns and allows uncertainty propagation into reliability assessments	Sensor replacement interrupts monitoring continuity; progressive degradation from ageing; temperature cycling, and mechanical effects remain difficult to fully eliminate	Time-dependent bias growing associated with sensor ageing, environmental exposure, and electronic degradation	Long-term sensor accuracy evaluation and baseline disruption studies [77]

**Table 5:** Cross-cutting taxonomy of computational methods reviewed in Section 3.

Method	Inferential Target	Likelihood	Data Mode	Parameter Type	Approximation	Forward-Model Cost
MCMC [113,127,128,129]	Static(*θ*)	Explicit	Batch	Static	Asymptotically exact	Moderate–High
SMC/Particle Filter [114,130]	Static(*θ*); Dynamic (*x*(*t*))	Explicit	Sequential	Static and state	Asymptotically exact	High
Variational Inference [131,132,133,134]	Static(*θ*)	Explicit	Batch/repeated	Static	Approximate (KL)	Low–Moderate
ABC [135,136,137]	Static(*θ*)	Implicit	Batch	Static	Approximate (tolerance)	Very High
BayesFlow/Neural SBI [138]	Static(*θ*)	Implicit	Batch (amortized)	Static	Approximate (neural)	Low (post-training)
Surrogate/Metamodel [139,140,141,142]	Forward model	N/A	Batch	N/A	Approximate	Low (post-training)
Deterministic Numerical Integration and Filtering [143]	Dynamic (*x*(*t*))	Explicit	Sequential	State	Exact (KF); Approx (EKF/UKF)	Low
Asymptotic/Laplace [144,145]	Static(*θ*)	Explicit	Batch	Static	Approximate (Gaussian)	Very Low
Hybrid Framework [65,115,146,147,148]	Coupling architecture	N/A	Batch	N/A	N/A	High

**Table 6:** Comparison of core posterior inference methods for sensor-informed bridge reliability assessment. Bridge Applications: demonstrated only; Best Suited For: prospective suitability.

Core Posterior Inference Methods	Computational Cost	Real-time/Online Suitability	Likelihood Function Required?	Non-Gaussian Posteriors?	Posterior Variance Accuracy	Marginal Likelihood Estimate	Demonstrated Reliability Index Suitability	Primary Limitation (Bridge)	Best Suited for	Convergence and Calibration	Bridge Applications
MCMC (Gibbs/MH/TMCMC)	High (serial sampling and repeated likelihood evaluations)	No	Yes	Yes	Full (rigorous uncertainty quantification)	TMCMC: Yes; Gibbs/MH: No	High; code compliance depends on limit state and model accuracy	Computationally intensive for high-fidelity FE models; not online	Offline batch updating; formal safety verification	Multiple-chain diagnostics; ESS	[65,67,149]
SMC/Particle Filter	Moderate (parallelizable over particles)	Yes	Yes	Yes	Full (complete posterior)	Available as byproduct)	High; code compliance depends on limit state and model accuracy	Cost grows with particle count and state dimension	Sequential updating where particle degeneracy and resampling cost are acceptable	Weight degeneracy; resampling threshold	[109,110,130,150]
VI	Low (deterministic optimization)	Yes (iterative batch; not sequential online)	Yes	Partial	Underestimated (mode-seeking KL divergence)	No	Limited (variance underestimation may give optimistic β)	Underestimates variance; constrains code-compliant thresholds	Large datasets; repeated inference across many scenarios	Variance and tail calibration; ELBO	[151]
ABC	Very high (extensive forward simulation budget required)	No	No	Yes	Approximate; depends on tolerance and summary statistics	No	Limited (posterior quality depends on summary statistic choice)	Very high simulation cost; sensitive to summary statistics and tolerance	Intractable likelihoods; offline analysis only	Tolerance sensitivity; summary sufficiency	None
Neural SBI (BayesFlow)	High offline training; near-zero online inference	Yes (post-training only)	No	Yes	Approximate; validated close to exact Bayesian in bridge application	No	Emerging (damage inference shown; formal *β* not yet demonstrated	No formal reliability index demonstrated; limited structural validation	Repeated inference after representative simulator training and distribution shift validation	Simulation gap; distribution shift	[137,138]

**Table 8:** Structured evidence synthesis of sensor-informed bridge reliability case studies reviewed. Sarmiento et al. [52] is the sole study explicitly reporting numerical β values. NR—Not Reported.

Study	Bridge	Sensor Type	Monitoring Duration	Reference Period	Assessment Target/Hazard/Limit State	Reported Uncertainty Reduction
Frangopol et al. [119]	Lehigh River SR-33, Pennsylvania, USA	Strain gauges	Not reported	NR	Flexural	Load-effect PDF updated; differs substantially from design
Ni et al. [64]	Tsing Ma Suspension Bridge, Hong Kong	Strain gauges	Long-term	NR	Fatigue	Stress-range PDF derived from measured data
Li et al. [121]	Long-span cable-stayed bridge	Strain gauges, accelerometers, WIM	Not reported	NR	Multi-hazard	Operational loading integrated across multiple hazards
Ni et al. [65]	Tsing Ma Suspension Bridge, Hong Kong	Strain gauges	Continuous	NR	Flexural	Posterior distributions evolved sequentially with data
Qu et al. [143]	Not specified	Stress sensors	Long-term	NR	Fatigue	Seasonal variability explicitly accounted for
Fan et al. [109,110]	Steel bridge	Strain gauges	Online	NR	Flexural	Real-time posterior updating via particle filter
Liu et al. [68]	Highway bridge, Wisconsin, USA	WIM	Not reported	NR	Flexural	Load-effect PDF derived from WIM data
Kwon et al. [164]	Steel bridges	Stress monitoring	Not reported	NR	Fatigue	Measured loading differs substantially from code-based
Jia et al. [51]	PSC bridge sections, Japan	Deflection sensors	Not reported	NR	Flexural	Resistance parameters updated; epistemic uncertainty reduced
Sarmiento et al. [52]	Kalix Bridge, Sweden (66 yr)	Prestress monitoring & material testing	Not reported	50 years	Serviceability	Prior *β* = 0.78, posterior *β* = 2.84; +2.06 reliability index units
Ponzo et al. [56]	Ponte della Musica, Rome, Italy	InSARDInSAR/SBAS	Multi-year archive	NR	Displacement	Thermal confounding identified; satellite displacement history established
Ghahari et al. [165]	Concrete bridge, USA	Accelerometers	Event-based	NR	Seismic	Soil–structure interaction uncertainties reduced
Maroni et al. [60]	Road bridges, Scotland, UK	Scour monitoring devices	Continuous	NR	Scour	Scour depth uncertainty significantly reduced at unmonitored locations

**Table 9:** Hierarchy of sensor-informed alarm levels in bridge reliability assessment.

Alarm Level	Decision Variable	Threshold	Reference Period	False-Alarm/Missed-Detection	Verification
Sensor/data quality [76]	Sensor health indicator; data completeness flag	Sensor-specific tolerance	Continuous	Low/high	Automated flag; engineer review
Response anomaly [25]	Posterior predictive anomaly index	Baseline deviation	Event-based	Moderate/moderate	Engineering investigation
Damage-state inference [99]	Posterior damage probability given observed data	Damage state threshold	Monitoring window	Moderate/high	Structural engineering review
Component limit-state [100]	Lower credible bound on *β*; upper credible bound on $P_{f}$	$\beta_{target}$; ${P_{f}}_{target}$	Code reference period	High/very high	Full reliability assessment
Network prioritization [170]	Reliability margin *β* − $\beta_{target}$	Network-relative ranking	Planning horizon	Network-scale	Asset management decision

## Data Availability

No new data were created or analyzed in this study. Data sharing is not applicable to this article.

## Abbreviations

The following abbreviations are used in this manuscript:

FORM	First-Order Reliability Method
SORM	Second-Order Reliability Method
SHM	Structural Health Monitoring
InSAR	Interferometric Synthetic Aperture Radar
POMDP	Partially Observable Markov Decision Process
RSL	Remaining Service Life
GNSS	Global Navigation Satellite System
FBG	Fiber Bragg Grating
PZT	Lead Zirconate Titanate (Piezoelectric Transducer)
FEM	Finite Element Method
DIC	Digital Image Correlation
WIM	Weigh-in-Motion
PSC	Prestressed Concrete
DFOS	Distributed Fibre Optic Sensors
DInSAR	Differential Interferometric Synthetic Aperture Radar
SBAS	Small Baseline Subset
MEMS	Micro-Electro-Mechanical Systems
PDF	Probability Density Function
FIM	Fisher Information Matrix
GAN	Generative Adversarial Network
WGAIN	Wasserstein Generative Adversarial Imputation Network
MNAR	Missing Not At Random
EMD	Empirical Mode Decomposition
VMD	Variational Mode Decomposition
OMA	Operational Modal Analysis
MODWT	Maximum Overlap Discrete Wavelet Transform
HZMB	Hong Kong–Zhuhai–Macao Bridge
ARIMA	AutoRegressive Integrated Moving Average
ROC	Receiver Operating Characteristic
CUSUM	Cumulative Sum Control Chart
EWMA	Exponentially Weighted Moving Average
SPRT	Sequential Probability Ratio Test
FDR	False Discovery Rate
GEV	Generalized Extreme Value
POT	Peaks-Over-Threshold
PCA	Principal Component Analysis
MCAR	Missing Completely At Random
GPR	Gaussian Process Regression
MAR	Missing At Random
BDR	Bayesian Dynamic Regression
GP	Gaussian Process
VoI	Value of Information
MCMC	Markov Chain Monte Carlo
SMC	Sequential Monte Carlo
VI	Variational Inference
ABC	Approximate Bayesian Computation
SBI	Neural Simulation-Based Inference
KF	Kalman Filter
EKF	Extended Kalman Filter
UKF	Unscented Kalman Filter
MH	Metropolis–Hastings
TMCMC	Transitional Markov Chain Monte Carlo
FOSM	First-Order Second Moment
KL	Kullback–Leibler
ELBO	Evidence Lower Bound
FE	Finite Element
MAP	Maximum A Posteriori
BUS	Bayesian Updating with Structural Reliability Methods
PHI2	PHI2 Method
MSR	Monitoring System Resolution
MSIM	Monitoring System Importance Measure
